# Whole-organ single-cell mapping defines growth dynamics and clonal organization in developing and adult zebrafish livers

**DOI:** 10.1371/journal.pbio.3004007

**Published:** 2026-09-28

**Authors:** Hsiao-Yuh Roan, Xuejiao Tian, Wei-Chen Chu, Chia-Ming Lee, Hsin Chen, Fiorency Santoso, Chung-Han Wang, Yu-Hsiu Liu, Uday Kumar, He-Yun Hsiao, Chiou-Hwa Yuh, Bi-Chang Chen, Chen-Hui Chen

**Affiliations:** 1 Institute of Cellular and Organismic Biology, Academia Sinica, Taipei, Taiwan; 2 Research Center for Applied Sciences, Academia Sinica, Taipei, Taiwan; 3 Nano Science and Technology Program, Taiwan International Graduate Program, Academia Sinica, Taipei, Taiwan; 4 Department of Engineering and System Science, National Tsing Hua University, Hsinchu, Taiwan; 5 Institute of Molecular and Genomic Medicine, National Health Research Institutes, Zhunan, Miaoli, Taiwan; University of Notre Dame, Center for Stem Cells and Regenerative Medicine, UNITED STATES OF AMERICA

## Abstract

Vertebrate organs undergo massive growth during the post-embryonic period. Yet, our understanding of how this organ-wide process is organized at single-cell resolution has been limited by an inability to monitor individual cells of different types throughout intact adult organs. Here we establish an integrated workflow of whole adult-organ expansion microscopy (WAO-ExM) that enables in toto single-cell-resolved visualization of every hepatocyte within a complete adult vertebrate liver. Using transgenic reporters to label hepatocyte nuclei, we quantified cell expansion dynamics across the entire post-embryonic growth period, finding that an intact adult liver spanning ~5 mm thickness had an average of 1,265,206 hepatocytes. The data further revealed a non-linear growth regimen in which cell number increased by 538-fold, with a temporally concentrated burst that was not reflective of overall body growth. Integration of lineage tracing with WAO-ExM revealed that cell number increases were driven by a few hepatocytes undergoing drastic clonal expansion at the whole-organ scale. Disruption of extracellular matrix laminins decoupled liver shaping from total cell number increases, suggesting independent regulatory control of tissue architecture and cell proliferation. We also utilized WAO-ExM to monitor diseased livers and other adult organs, including heart and pancreas. Altogether, these findings bridge micrometer-scale cell behaviors with centimeter-scale organ growth, and establish a generalizable platform for adult vertebrate organs to be fully resolved at bona fide single-cell resolution.

## Abstract 2

Vertebrate organs undergo massive growth during the post-embryonic period, but it remains unclear how organs are organised at single-cell resolution. This study develops whole adult-organ expansion microscopy (WAO-ExM) to visualize every single hepatocyte in the zebrafish liver during post-embryonic growth.

## Introduction

Vertebrate organs, such as liver and heart, grow drastically during the post-embryonic period. This growth is characterized by a marked increase in cell number alongside distinct changes in organ size and shape. While early embryonic organ morphogenesis can be examined at both cellular and organ levels in small vertebrate models such as zebrafish, it remains technically challenging to capture individual cell behaviors across entire organs at later developmental stages. In particular, limited optical translucence, together with the difficulty of conducting cell-resolution volumetric imaging in thick tissue samples exceeding ~100 µm has hindered in-depth studies of organ morphogenesis during larval-to-adult transitions [1]. To the best of our knowledge, quantitative assessments have not been made of the cell number expansion required for growth of an adult vertebrate organ to its final millimeter- to centimeter-scale size and shape. Similarly, it is unknown how cell proliferation is spatiotemporally coordinated with organ morphology and function. Methods that provide both bona fide single-cell resolution and a complete, organ-wide view across many thousands of cells are critically needed to adequately address these questions.

The combination of expansion microscopy (ExM) with lightsheet microscopy has emerged as a powerful method for imaging subcellular structures in large biological specimens, including dendritic spines and neuronal projections across the mouse cortex and whole fly brains [2–7]. ExM can create optically transparent tissues by physically and isotropically expanding the specimen, thereby reducing light scattering and enabling optical imaging of fine structures, including subsynaptic protein organization in larval zebrafish [8]. However, expansion in all three spatial dimensions also markedly increases the size of a sample. This change in sample size poses practical challenges for conventional confocal microscopy, including greatly prolonged acquisition time, photobleaching, and limitations of objective working distances. When integrated with macro-scale imaging optics, lightsheet microscopy can effectively overcome these limitations. For instance, this combined approach can readily enable optical imaging of individual cells and neurons across the entire expanded, centimeter-scale postnatal mouse brain (up to 5 × 2.4 × 3.7 cm^3^) at an effective resolution of ~300 nm [6,9–11]. Despite these advances, such approaches have not yet been applied to longitudinally resolve the developmental trajectories of adult vertebrate organs at whole-organ scale with both single-cell resolution and cell-type specificity. Thus, the full potential of these methods to quantitatively define how cellular behaviors scale to drive organ growth remains to be realized.

Among vertebrate organs, the liver is the largest solid organ and likely contains the highest total number of cells in the adult state. The tissue comprises multiple cell types, including hepatocytes, cholangiocytes, sinusoidal endothelial cells, Kupffer cells, and fibroblasts [12,13]. Hepatocytes are the principal functional cells in the liver and constitute up to 80% of its mass; these cells carry out essential metabolic, endocrine, secretory, and detoxification functions [12]. Using the zebrafish model, Gao and Peng and colleagues reported that liver growth is predominately driven by hepatocyte proliferation during the post-embryonic period [14]. The hepatocyte number was estimated to increase by ~900-fold from larval to adult stages, based on measurements of overall liver volume [14]. Intriguingly, Unterweger and Ober’s group utilized whole-mount imaging and cell lineage-tracing techniques to further reveal that individual hepatocyte progenitors contribute heterogeneously to this growth [15]. Some hepatocytes generate giant, dominant clones that occupy substantial portions of the ventral lobe by adulthood [15]. This profound finding suggests that the massive proliferation of a few, select progenitor/stem cells may serve as a key cellular growth mechanism that mediates post-embryonic expansion of essential vertebrate organs, including the heart, skin epidermis, and skeletal muscle [16–18]. Notably, cell growth dynamics must be viewed at a whole-organ level and with sufficient resolution in order to observe this conserved, intricate mechanism, which might otherwise remain hidden.

Here, we developed WAO-ExM, aiming to visualize each and every hepatocyte in the zebrafish liver across representative stages of post-embryonic growth period. By integrating tissue clearing, expansion, axicon-based Bessel beam lightsheet microscopy, and long working distance excitation and detection objectives, we were able to achieve a whole-liver view of individual hepatocyte nuclear signals spanning up to 8.6 × 16.3 × 4.8 mm^3^ in three-dimensional expanded sample space. We determined that the hepatocyte number increases from 2,352 at the larval stage [7 days post-fertilization (dpf)] to an average of 1,265,206 cells in adult (3–6 months of age) livers. When used in concert with a multicolor-based cell lineage-tracing tool, WAO-ExM readily captured the emergence of hepatocyte-dominant clones in the ventral lobe, facilitating quantitative determination of the clone sizes and cell numbers within. Furthermore, we performed genetic, temporal perturbation of the extracellular matrix component *lamb1a* during the post-embryonic period. These experiments revealed that liver lobe morphogenesis was severely compromised, yet total hepatocyte number was not changed, suggesting that macroscopic organ shaping and microscopic cell proliferation are governed by independent mechanisms. Finally, we demonstrated that the platform can be readily applied to determine the exact numbers of hepatocytes in cancerous liver, as well as key cell types in major adult organs, including epicardial cells and pancreatic beta cells. This study not only defines the intricate cellular dynamics underlying organ-level liver growth, but it also establishes a robust framework for resolving adult vertebrate organ morphogenesis with single-cell and cell-type-specific precision.

## Results

### In toto visualization of hepatocytes within a complete adult liver

To visualize every hepatocyte in the adult zebrafish liver, we first generated a stable transgenic line using the 2.8 kb *fabp1a* promoter [19] to fluorescently label each hepatocyte’s nucleus and membrane [*Tg(fabp1a:H2A-mCherry; fabp1a:palm-mTurquoise*); S1A Fig]. We then verified that the line has consistent labeling of hepatocytes throughout the post-embryonic period and adult stages (S1A and S1B Fig). Through a systematic survey of 7,115–52,015 hepatocytes from individuals at 7 dpf, 21 dpf, 42 dpf, and 3–6-month-old adults, we found that although the majority of hepatocytes (~99%) are mononucleated, a subtle yet progressive increase in nuclear heterogeneity was evident during post-embryonic growth (S1C Fig). Of note, this finding is consistent with a previous report [15]. Intriguingly, individual hepatocyte volume also increased by an average of 1.75-fold over the course of the larva-to-adult transition (S1D Fig), presumably reflecting functional maturation of these cells. Due to light scattering and poor optical resolution along the z-axis, we could only survey a small number of hepatocytes located near the tissue periphery, within ~100 µm of depth. This optical limitation represents a common technical hurdle for studying cell dynamics in post-embryonic zebrafish tissues and organs. Thus, we set out to explore a combined ExM and Bessel beam lightsheet approach to enable centimeter-scale, organ-level visualization of all constituent cells.

Using the CLARITY clearing method followed by a potassium acrylate-based hydrogel-mediated tissue swelling step [2,20], we readily expanded adult zebrafish liver by 4-fold (corresponding to an approximately 64-fold increase in volume). In addition, the whole organ was rendered optically transparent (Fig 1A; see Materials and methods for details). The expanded, intact liver embedded in hydrogel reached a size that was no longer compatible with conventional lightsheet microscopy (i.e., 9.6 × 21.2 × 5.3 mm^3^), so we performed volumetric imaging using Bessel beam lightsheet microscopy. We acquired 6 × 15 tiles with 2,443 z-steps over the course of about 5 hours. Imaging was conducted at a voxel resolution of approximately 0.81 × 0.81 × 2.45 µm^3^, corresponding to an effective resolution of about 200 × 200 × 600 nm^3^ after 4 × expansion. Each adult liver yielded a volumetric dataset of approximately 1.7 TB (Fig 1B and 1C). Of note, the z-step number varied depending on the sample/gel thickness, and adjacent tiles included a 20% overlap for stitching (see Materials and methods for further details). Notably, this setup enabled us to acquire unambiguous, single-cell resolution images of individual hepatocyte throughout the entire three-lobed adult liver, regardless of its position relative to the detection objective (Fig 1D and S1 Movie). We noted that compared with cleared but non-expanded tissues, the expanded tissues had a marked improvement in z-axial resolution (Fig 1E; compare S1 and S2 Movies), facilitating precise three-dimensional enumeration of each and every hepatocyte. Together, these results demonstrate that the approach, which we term WAO-ExM (Whole Adult-Organ Expansion Microscopy), enables simultaneous capture of overall liver morphology and accurate cell counts across the entire adult organ.

**Fig 1:**
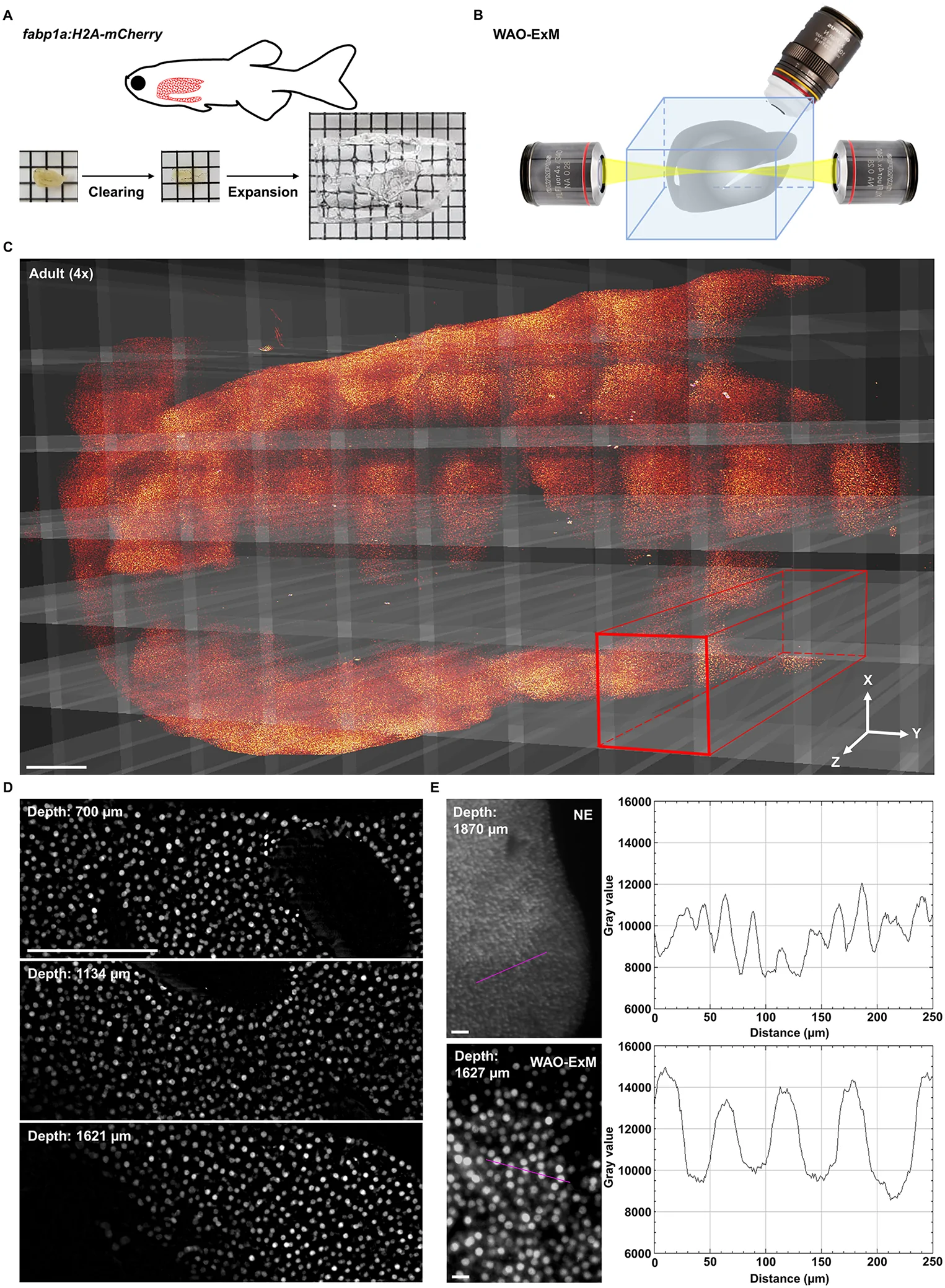
In toto visualization of each individual hepatocyte within a complete adult liver. **(A)** Bright-field images showing tissue clearing and isotropic expansion of adult *Tg(fabp1a:H2A-mCherry)* liver samples. Grid squares are 0.25 mm × 0.25 mm. **(B)** Schematic of whole-adult-liver volumetric imaging, highlighting the z-axis tilt-free scanning strategy. The imaging system comprises a pair of excitation objectives (0.28 NA, 28.8-30.0 mm WD) and a detection objective (XLPLN10XSVMP, 0.6 NA, 8 mm WD). **(C)** Whole adult-organ expansion microscopy (WAO-ExM) image of a 4× expanded adult liver, showing nuclei visualized by glow rendering (stitched). The red rectangle box marks a single tile. A total of 6 × 15 tiles and 2,443 z-steps were acquired for each adult liver. Scale bar, 1 mm. **(D)** Representative optical sections acquired from superficial (700 µm) to deep (1,134 µm and 1,621 µm) regions of the liver, highlighting the preservation of z-axis resolution throughout the sample depth. Images were gamma-corrected for improved visualization. Scale bar, 500 µm. **(E)** Comparison of z-axis resolution in non-expanded (NE; imaging depth, 1,870 µm) and WAO-ExM-expanded (4×; imaging depth, 1,627 µm) liver samples, together with signal-to-background ratio measurements. Purple lines denote the region used for signal intensity measurements. Scale bars, 50 µm. Source data are available in S1 Data.

### Hepatocyte number reaches an average of 1.27 million cells by adult stages

To determine the extent of increase in hepatocyte numbers throughout the post-embryonic growth period, we applied WAO-ExM to visualize individual hepatocyte nuclei in samples collected at 7, 21 and 42 dpf, as well as 3–6-month-old adults (Fig 2A and 2B). Of note, the liver is too small to be dissected as an independent unit at 7 and 21 dpf, so we applied WAO-ExM to whole animals or body trunk pieces to preserve organ integrity during the clearing and expansion steps. It is also worth noting that despite the presence of rigid skeletal structures (e.g., skull and vertebral column), the entire zebrafish larva could be readily and isotropically expanded up to 10-fold (S2A Fig), allowing for subcellular, submicrometer-resolution imaging of individual nuclear chromosomal patterns (S1B and S1C Fig; S3 Movie). These results highlight the potential of the expansion and imaging approach to facilitate high-resolution, volumetric optical imaging of diverse internal organs and structures beyond the liver and throughout the entire course of post-embryonic zebrafish development.

**Fig 2:**
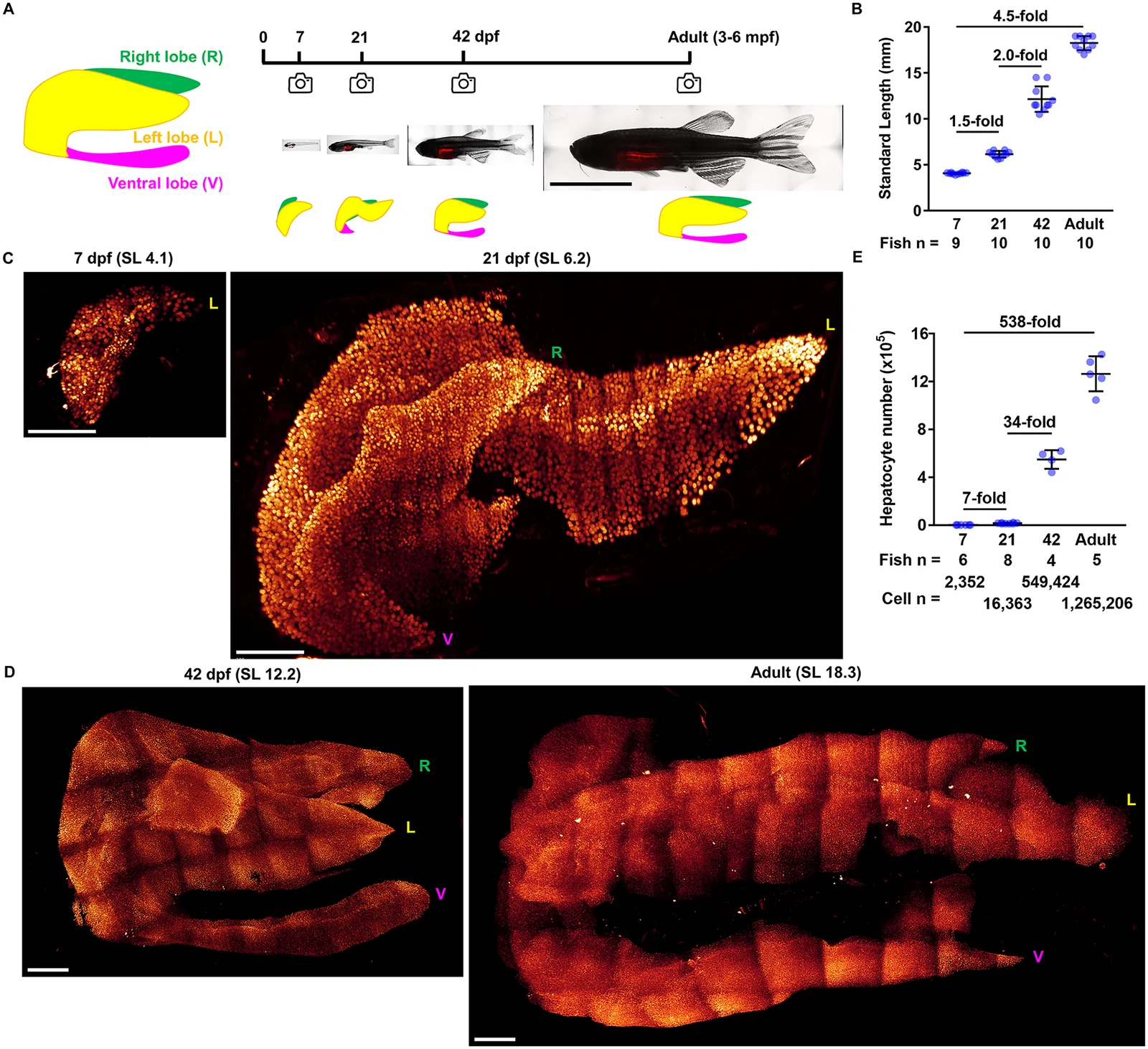
An adult liver contains approximately 1.27 million hepatocytes. **(A)** Developmental timeline and liver images captured at 7, 21, and 42 dpf, and in adults (3-6 mpf). Stitched whole-mount epifluorescence images of *Tg(fabp1a:H2A-mCherry)* fish are presented alongside schematic illustrations of the corresponding liver morphology. Liver lobes are color-coded as left (L, yellow), right (R, green), and ventral (V, magenta). Scale bar, 1 cm. **(B)** Quantification of Standard Length across developmental stages. Data are shown as mean ± s.d.; the number of fish analyzed is indicated below the x-axis. Fold changes are shown above the indicated time points. **(C, D)** WAO-ExM images (glow rendering) of livers expanded 10× at 7 and 21 dpf, and 4× at 42 dpf and adulthood (stitched). The adult liver image shown here is the same representative sample presented in Fig 1C, but viewed from a different angle. Liver lobes are indicated by color-coded labels: left (L, yellow), right (R, green), and ventral (V, magenta). SL, Standard Length in millimeters, shown as the mean value. Scale bar, 1 mm. **(E)** Quantification of total hepatocyte number across developmental stages. Data are shown as mean ± s.d.; the number of fish examined is indicated below the x-axis, and the annotated cell numbers are the mean values. Fold changes are shown above the indicated time points. Source data are available in S1 Data.

Using WAO-ExM, we determined that the hepatocyte numbers increased drastically (538-fold) during the larval-to-adulthood transition. The total hepatocyte number rose from 2,352 at 7 dpf to an average of 1,265,206 cells per adult individual (*n* = 4–8, respectively; Fig 2C–2E; S1–S4 Movies). Intriguingly, we found that while the body Standard Length increased by only ~2-fold between 21 and 42 dpf, total hepatocyte numbers surged by ~34-fold during the same three-week period (Fig 2B and 2E). We therefore considered this period a critical phase of massive liver growth that is not apparent from external whole-body appearance. Because our measurements were obtained at discrete developmental time points, the precise onset, and duration of this accelerated growth remain to be determined through finer temporal sampling. Nonetheless, by defining the absolute number of hepatocytes in a growing individual, we demonstrated that liver growth is not gradual but instead accelerates markedly within a three-week developmental window.

### The liver growth burst coincides with the emergence of dominant hepatocyte clones

To determine the proliferation potential of individual hepatocytes during this critical phase of liver growth, we generated a Brainbow-based [21] transgenic line and an inducible Cre line using a dually inducible cassette [22]. This transgenic design enabled long-term tracking of individual hepatocytes and their clonal descendants throughout the post-embryonic growth period (Fig 3A and 3B). The double transgenic line [*Tg(fabp1a:brainbow; fabp1a:iCre*)] is hereafter referred to as *liverbow*. In this line, we detected no leaky Cre activity at 7 dpf, 42 dpf, or adult stages (*n* = 13–18 individuals per stage; Figs 3C, S3A, and S3B). In contrast, the addition of doxycycline and tamoxifen at 4 dpf led to robust induction of multicolor barcoding of individual larval hepatocytes by 8 dpf (Fig 3C). Remarkably, the emergence of dominant hepatocyte clones in both juvenile and adult livers was highly evident, particularly in the ventral lobe and distal tip regions (Fig 3D–3F). These clones were readily captured in 34 of the 35 adult livers examined, with representative images shown in Figs 3E, 3F, and S3C. These findings were in line with previous lineage-tracing studies of embryonic hepatocyte progenitors [15]. To determine what portion of the liver could be populated by a dominant clone, we titrated down Cre activity such that only a few hepatocytes were labeled. Then, we applied WAO-ExM to the ventral lobes that contained just one single labeled hepatocyte clone (Fig 3G). By surveying four dominant clones in three-dimensional space, we determined that a single hepatocyte labeled at 4 dpf could generate up to 24.9% of the entire ventral lobe by 42 dpf; this proportion corresponded to 12,421 cells based on DAPI-positive nuclei contained within the clone-occupied volume (12.7% and 6,602 cells on average; Fig 3H and 3I; S6 Movie). Of note, the calculated cell number does not represent an exact count of hepatocyte descendants in this context, and is likely an overestimate, as nuclei from interstitial and perivascular cells may also be included within the quantified domain. Also, although both standard and reduced Cre activity generated dominant clones of similar sizes, we cannot completely exclude the possibility that some color-labeled territories comprise neighboring clones of similar colors. Nevertheless, our ability to take a whole-organ, three-dimensional view of individual hepatocyte growth profiles allowed us to conclude that like embryonic hepatocyte progenitors, postembryonic hepatocytes are not equally proliferative. Instead, a subset of cells selectively and dramatically expands in a spatially restrictive manner, likely accounting for the sharp burst in hepatocyte number over a short time period.

**Fig 3:**
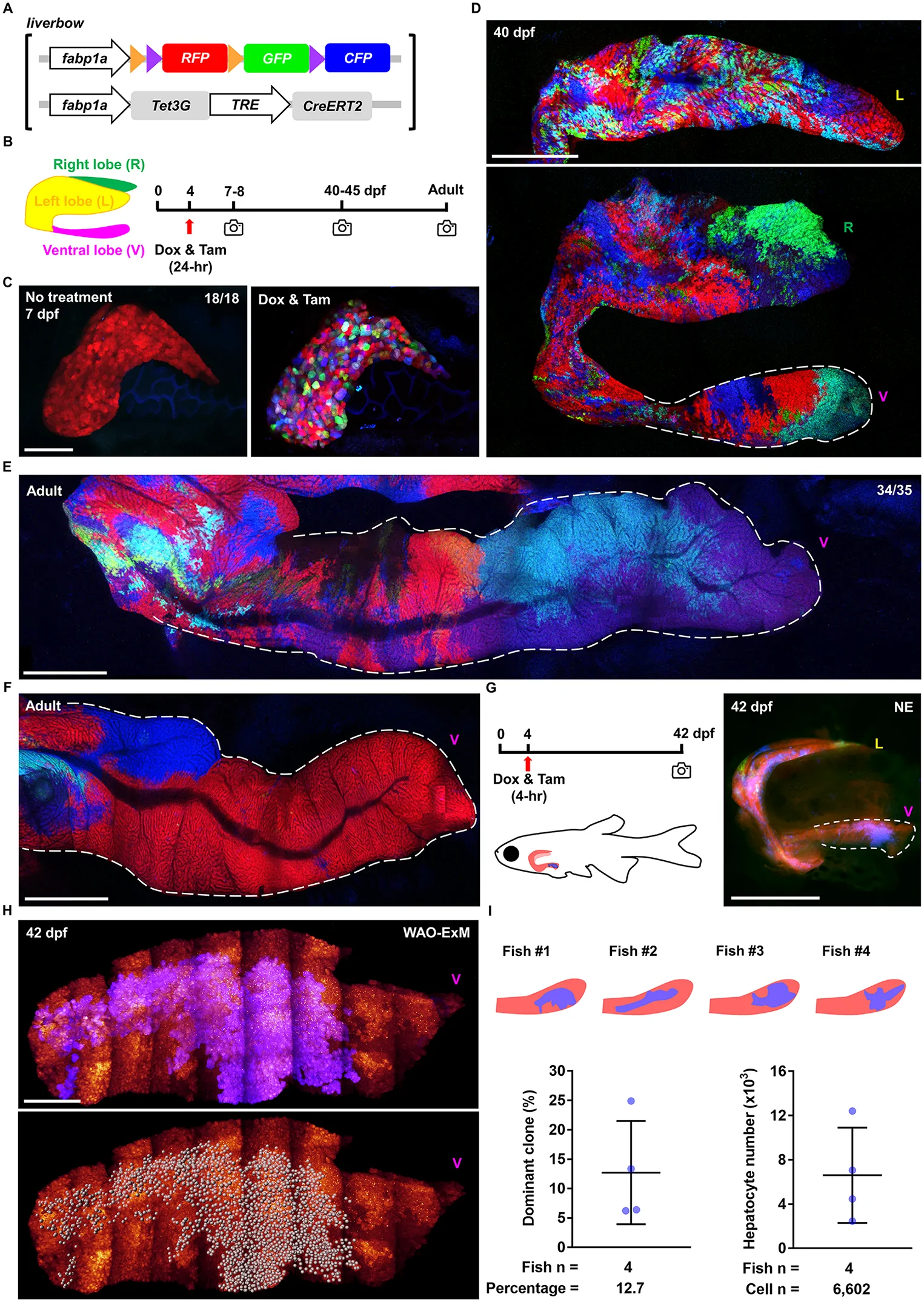
In toto monitoring of individual cell growth captures dominant hepatocyte clones. **(A)** Schematic of the *liverbow* transgenic constructs. **(B)** Experimental timeline and liver collection at 7/8 dpf, 42 dpf, and adult (3–6 mpf) stages. Doxycycline (Dox) and tamoxifen (Tam) were administered at 4 dpf for 24 hours to activate Cre recombinase and induce multicolor-barcoding of hepatocytes. **(C)** Confocal images of livers at 7/8 dpf livers in the absence or presence of doxycycline and tamoxifen treatment (Dox & Tam). Of note, no leaky recombination was detected in any of the 18 individuals examined. Scale bar, 100 µm. **(D)** Confocal images of livers showing hepatocyte clonal growth at 40 dpf (stitched). White dashed line delineates the boundary of the ventral lobe. Liver lobes are indicated by color-coded labels: left (L, yellow), right (R, green), and ventral (V, magenta). Scale bar, 500 µm. **(E)** Confocal image of an adult ventral liver lobe with multiple dominant hepatocyte clones (stitched). White dashed line delineates the ventral lobe boundary (V, magenta). Scale bar, 500 µm. **(F)** Confocal image of an adult ventral liver lobe containing a single dominant labeled hepatocyte clone (stitched). White dashed line delineates the ventral lobe boundary (V, magenta). Scale bar, 500 µm. **(G)** Experimental timeline and liver collection at 42 dpf. Dox and Tam were administered at 4 dpf for 4 hours to induce sparse labeling of hepatocyte clones. (Right) An isolated unexpanded liver (NE) showing a dominant hepatocyte clone in the ventral lobe. White dashed line marks the ventral lobe boundary. Scale bar, 500 µm. **(H)** WAO-ExM images of a 42 dpf ventral liver lobe expanded 4× (stitched). A single labeled hepatocyte clone is shown in blue (Top), and the same clone is overlaid with nuclear signal (bottom). Scale bar, 500 µm. **(I)** Quantification of the individual dominant clone volume as a fraction of total ventral lobe volume (left) and the estimated number of cells contained within each clone volume (right). Data are shown as mean ± s.d.; the number of fish examined is indicated below the x-axis, and the annotated cell numbers are mean values. Source data are available in S1 Data.

### Laminin activity primarily affects liver shape rather than total cell number

The extracellular matrix (ECM) component laminins are reported to be highly expressed in the developing liver [23] and have diverse, seemingly conflicting functions on hepatocyte proliferation [24–27]. Therefore, we sought to investigate the influence of laminins on post-embryonic liver growth using WAO-ExM. Using RT-qPCR, we first determined that several ECM components, including laminins (*lamb1a* and *lamc1*) and key growth-regulating factors (*lef1* and *ddx52*), were expressed at much higher levels in the livers of 28 dpf larval zebrafish than in adults (S4A Fig). We then took advantage of the temperature-sensitive *lamb1a*^*sde1*^ mutant allele [28] to achieve temporally controlled real-time inactivation of laminin in live zebrafish, particularly during the post-embryonic, burst-growth period (Fig 4A and 4B). Of note, *lamb1a* inactivation alone is sufficient to disrupt laminin complex deposition in the basement membrane [28]. Three weeks of *lamb1a* inactivation had no apparent effect on overall fish growth, as assessed by Standard Length, despite the presence of previously described fin degradation phenotypes [28] (Fig 4B and 4C). Nevertheless, approximately one-third of the mutant fish developed severe liver morphological defects (*n* = 13 out of 38; S4B Fig), including complete loss of a major lobe (4 out of 13) or disproportionate lobe growth (9 out of 13). In contrast, no such defects were detected in any heterozygous siblings (*n* = 37 out of 37). Intriguingly, using WAO-ExM, we found that, despite these pronounced morphological defects, the total hepatocyte number did not differ significantly between sibling controls and deformed mutant livers in this limited dataset (195,242 versus 187,970 cells, respectively; estimated mean difference, −7,273 ± 36,802 cells; 95% CI, −96,101–81,556; Fig 4D–4F; the four mutant fish analyzed by WAO-ExM were the same morphologically affected animals shown in S4B Fig). Although our *lamb1a* perturbation was systemic rather than liver-specific, and its effects may have been indirect, potentially resulting from altered gut morphogenesis [15], this unexpected finding suggested that laminins may primarily influence liver growth and development by modulating the overall organ shaping process rather than by controlling hepatocyte proliferation. It is worth noting that without WAO-ExM, we would not have been able to decouple the macroscopic, organ-level defects from microscopic changes in the exact total cell number within the organ.

**Fig 4:**
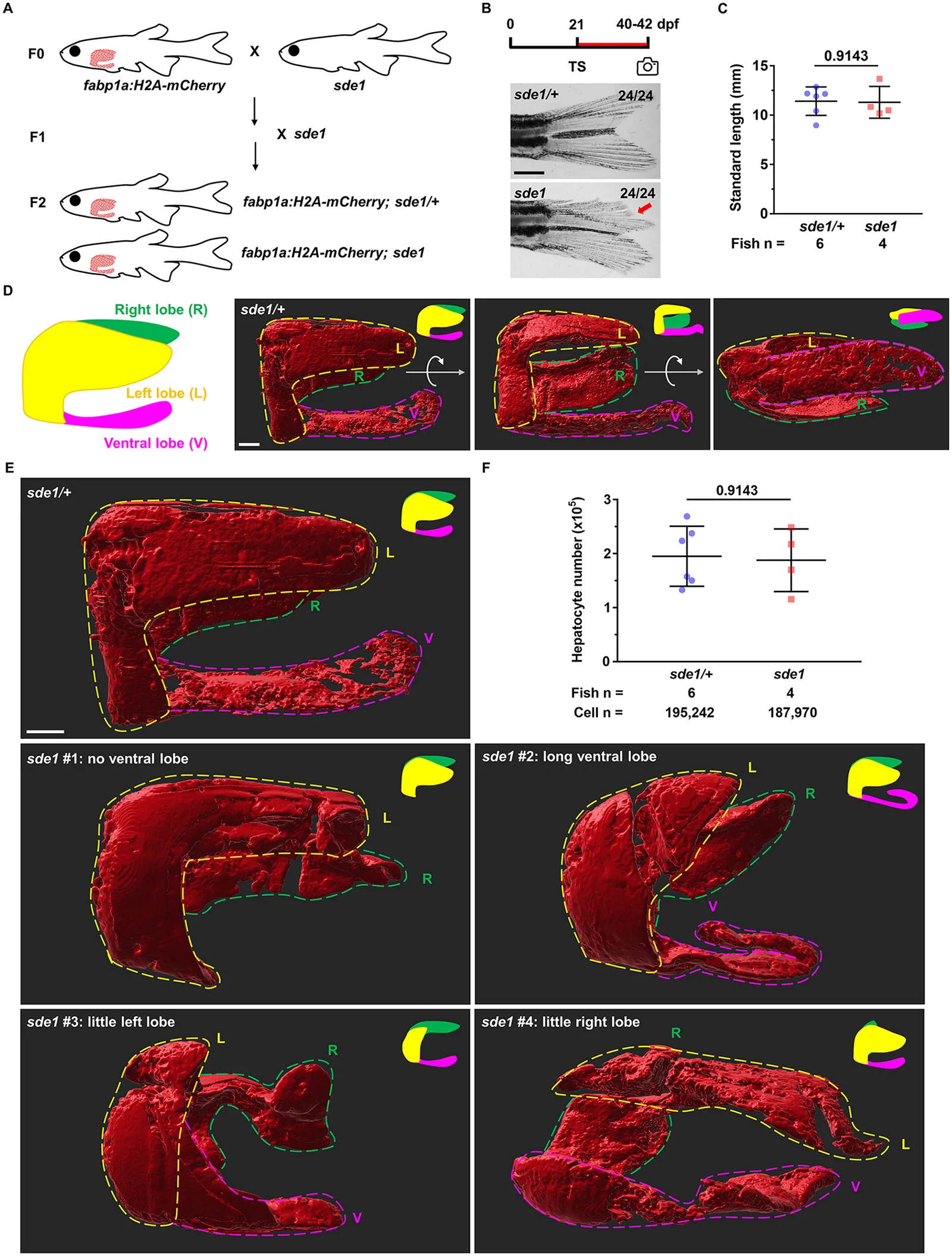
Laminin activity governs liver growth by defining its shape rather than its total cell number. **(A)** Mating scheme for introducing the temperature-sensitive *lamb1a*^*sde1*^ allele into the *Tg(fabp1a:H2A-mCherry)* background. **(B)** Experimental design for temporal inactivation of *lamb1a* by temperature shift (TS; red bar indicates 34 °C incubation). Bright-field images show the fin atrophy phenotype in *sde1* mutants; the red arrow marks degenerated fin tissue. Scale bar, 1 mm. **(C)** Quantification of Standard Length in control (*sde1/+*) and mutant (*sde1*) fish. Data are shown as mean ± s.d.; the number of fish examined is indicated below the x-axis. Statistical significance was assessed using a two-tailed Mann–Whitney test. **(D)** Three rotational views of a control (*sde1/+*) liver. Individual liver lobes are indicated by color-coded labels: left (L, yellow), right (R, green), and ventral (V, magenta). Three-dimensional reconstructions were generated from whole-liver WAO-ExM datasets using the Imaris surface-rendering function (red). Scale bar, 1 mm. **(E)** Representative whole-liver morphologies of control (*sde1/+*) and *lamb1a*-affected (*sde1*) fish, reconstructed using the Imaris surface-rendering function (red). Liver lobes are indicated by color-coded labels: left (L, yellow), right (R, green), and ventral (V, magenta). Scale bar, 1 mm. **(F)** Quantification of total hepatocyte number in control (*sde1/+*) and mutant (*sde1*) fish. Data are shown as mean ± s.d.; the number of fish examined is indicated below the x-axis, and the annotated cell numbers are mean values. Statistical significance was assessed using a two-tailed Mann-Whitney test. The resulting p-value is identical to that shown in Fig 4C. Of note, the four mutants analyzed by WAO-ExM here were the same morphologically affected animals shown in S4B Fig. Source data are available in S1 Data.

### In toto visualization of cancerous liver, heart, and pancreas in adult zebrafish

To determine if WAO-ExM may be applicable for studies on disease, we applied the method to examine total hepatocyte number in an established zebrafish liver cancer model (Fig 5A). Transgenic *Tg(fabp10a:tert; p53− /−)* fish with liver-specific over-expression of telomerase reverse transcriptase in a *p53* mutant background are reported to develop classical hallmarks of hepatoma at both cellular and molecular levels by 30 dpf [29]. We first noted that, at 21 dpf, the transgenic fish exhibited signs of developmental delay, as indicated by the bending angle of the posterior notochord [1] (Fig 5A) and a moderate reduction in Standard Length (~16%; Fig 5B). Intriguingly, WAO-ExM revealed that these previously established cellular-level histological abnormalities were accompanied by a 66% lower number of reporter-positive hepatocyte nuclei in transgenic animals than in age-matched wild-type controls (23,049 versus 7,769; *n* = 3 and 4, respectively; Fig 5C and 5D). Although preliminary and seemingly counterintuitive, given that cancerous tissues are often associated with increased cell proliferation, these findings demonstrate the potential of WAO-ExM to quantify organ-wide cellular alterations in diseased tissue. Meanwhile, it is important to note that the lower number of reporter-positive hepatocyte nuclei could reflect developmental delay, altered hepatocyte differentiation, cell death, or reduced or heterogeneous expression of the *fabp1a:H2A-mCherry* reporter in transformed hepatocytes. Further experiments will be required to distinguish among these possibilities.

**Fig 5:**
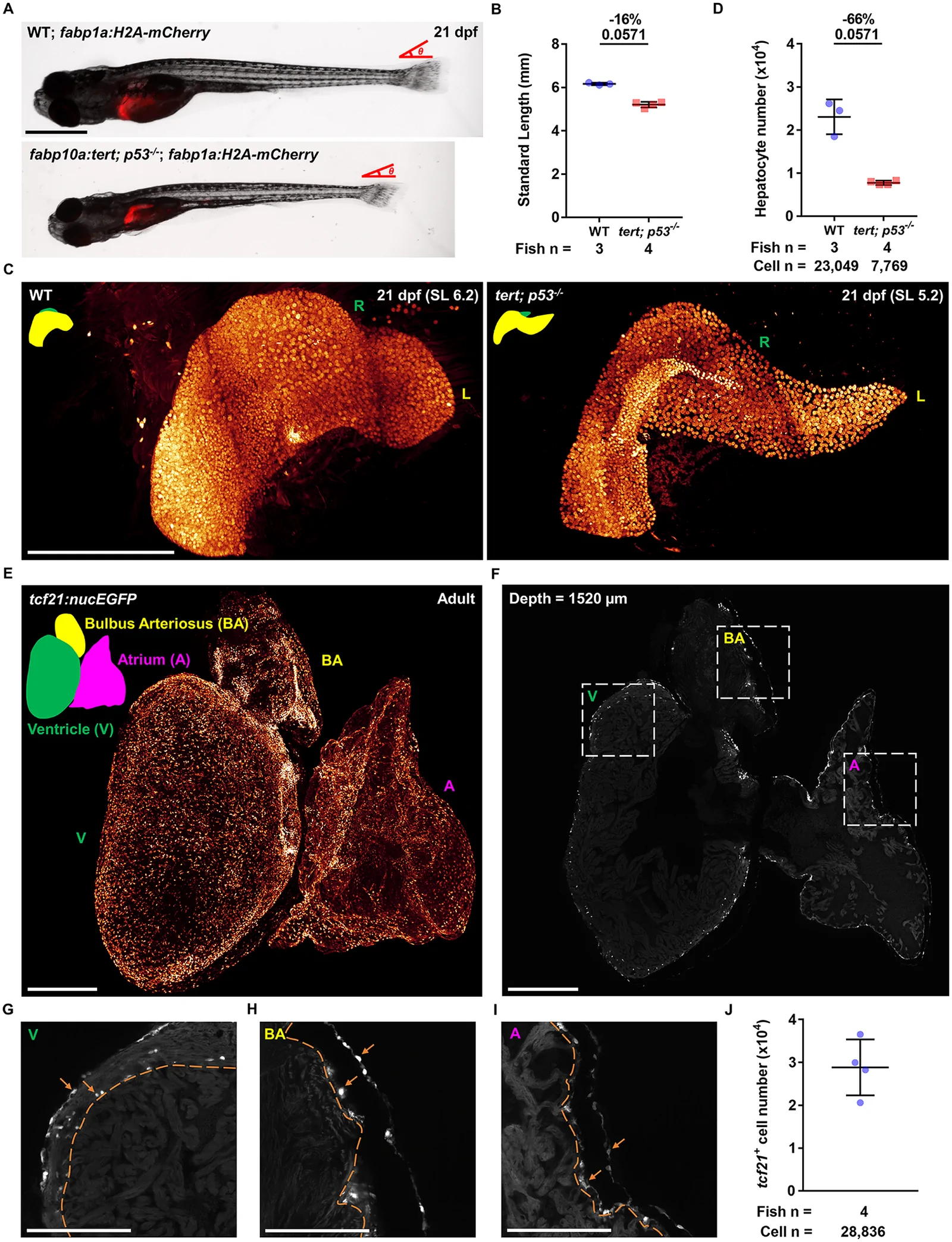
In toto visualization of all cells in cancerous liver and epicardial cells in adult zebrafish. **(A)** Whole-mount epifluorescence images of 21 dpf wild-type (WT) control and cancer-prone (*fabp10a:tert; p53*^*−/−*^) fish. Red lines indicate the bending angle of the posterior notochord. Scale bar, 1 mm. Scale bar, 1 mm. **(B)** Quantification of Standard Length in 21 dpf WT and cancer-prone fish. Data are shown as mean ± s.d.; fish numbers are indicated below the x-axis. Statistical significance was assessed using a two-tailed Mann-Whitney test. **(C)** WAO-ExM images of 10× expanded 21 dpf WT control and cancerous livers, alongside a color-coded labels indicating the left (L, yellow) and right (R, green) liver lobes. SL, Standard Length in millimeters, shown as the mean value. Scale bar, 1 mm. **(D)** Quantification of total hepatocyte number in WT and cancerous livers. Data are shown as mean ± s.d.; the number of fish examined is indicated below the x-axis, and the annotated cell numbers are mean values. Statistical significance was assessed using a two-tailed Mann-Whitney test. **(E)** WAO-ExM image of a 4× expanded adult heart, showing *Tg(tcf21:nuceGFP)*-labeled epicardial cells. Schematic marks the three cardiac compartments: bulbus arteriosus (BA, yellow), ventricle (V, green), and atrium (A, magenta). Scale bar, 1 mm. **(F)** WAO-ExM section of an adult heart showing the distribution of epicardial cells and bilayered organization across cardiac compartments. White dashed boxes indicate regions shown at higher magnification in (G-I). Scale bar, 1 mm. **(G–I)** High-magnification views of the (G) ventricle, (H) bulbus arteriosus, and (I) atrium regions indicated in **F.** Orange dashed lines mark the epicardium-myocardium boundary, and arrows highlight epicardial cells located in different epicardial layers. Scale bars, 500 µm. **(J)** Quantification of epicardial cell number in adult hearts. Data are presented as mean ± s.d.; the number of fish examined is indicated below the x-axis, and the annotated cell numbers are mean values. Source data are available in S1 Data.

To assess whether WAO-ExM may be used to study adult zebrafish organs beyond the liver, we applied the method to visualize each and every epicardial cell covering the heart (Fig 5E). In previous studies, epicardial cells have been shown to play a pivotal role in supporting cardiomyocyte proliferation and heart regeneration [30,31]. Using WAO-ExM, we determined that the adult heart contains an average of 28,836 *tcf21*-postitive epicardial cells enclosing the ventricle, atrium, and bulbus arteriosus (Fig 5E–5J; S7 Movie). Consistent with prior studies [32], we captured the bilayered arrangement of epicardial and epicardial-derived cells covering the adult ventricular wall (Fig 5F). Moreover, the in toto visualization of the heart at both organ and cellular levels allowed us to further determine that the bilayered arrangement is not limited to the ventricle. The arrangement also extends to support and enclose the entire adult atrium and bulbus arteriosus (Fig 5G–5I; S7 Movie). This finding has implications not only regarding the structural roles and functions of these critical cells across distinct cardiac compartments, but it also highlights the unique capability of WAO-ExM to complement histological assays, which are restricted to two-dimensional cellular information from limited tissue regions. In addition to its utility for studying solid organs (i.e., liver and heart), WAO-ExM can also be applied to visualize and precisely enumerate cells within endocrine organs, such as pancreatic beta cells (3,152 ins-positive cells on average in the primary islet of an adult individual; S5A–S5C Fig); it also has sufficient subcellular resolution to reveal nuclear features (S5B Fig; S8 Movie). Altogether, our findings lead us to conclude that WAO-ExM permits accurate, whole-organ quantification of specific cell populations across both solid and endocrine organs. In addition, it offers a unique framework for interrogating organ-wide growth dynamics and cellular organizational principles that may be missed by local sampling or conventional histological approaches.

## Discussion

Here, we developed an in toto imaging platform and analysis pipeline (WAO-ExM; Fig 6) that enables thorough examination of adult vertebrate organs in their native, intact state across centimeter-scale volumes with single-cell resolution and cell-type specificity. Using this framework, we visualized every hepatocyte in developing and adult livers from individual animals, and thus determined the exact number of cells present as the organ acquires its adult size and shape. By combining this in toto system with cell lineage-tracing techniques, we further captured and defined the contributions of emerging dominant hepatocyte clones in three-dimensional space. Our use of genetic tools to perturb ECM activity revealed that the liver shaping process and amount of hepatocyte expansion are not intrinsically coupled, but are independently regulated. Finally, we demonstrated that the platform can be readily applied to disease contexts and other adult organs to uncover organ-level, cellular organizational patterns that only become evident when both macroscopic and microscopic details are simultaneously resolved. Thus, we propose that use of WAO-ExM with the zebrafish model can fill a critical technical gap in studies on organismal development and adult organ morphogenesis, where imaging approaches typically lack an organ-wide perspective or sufficient cellular resolution.

**Fig 6:**
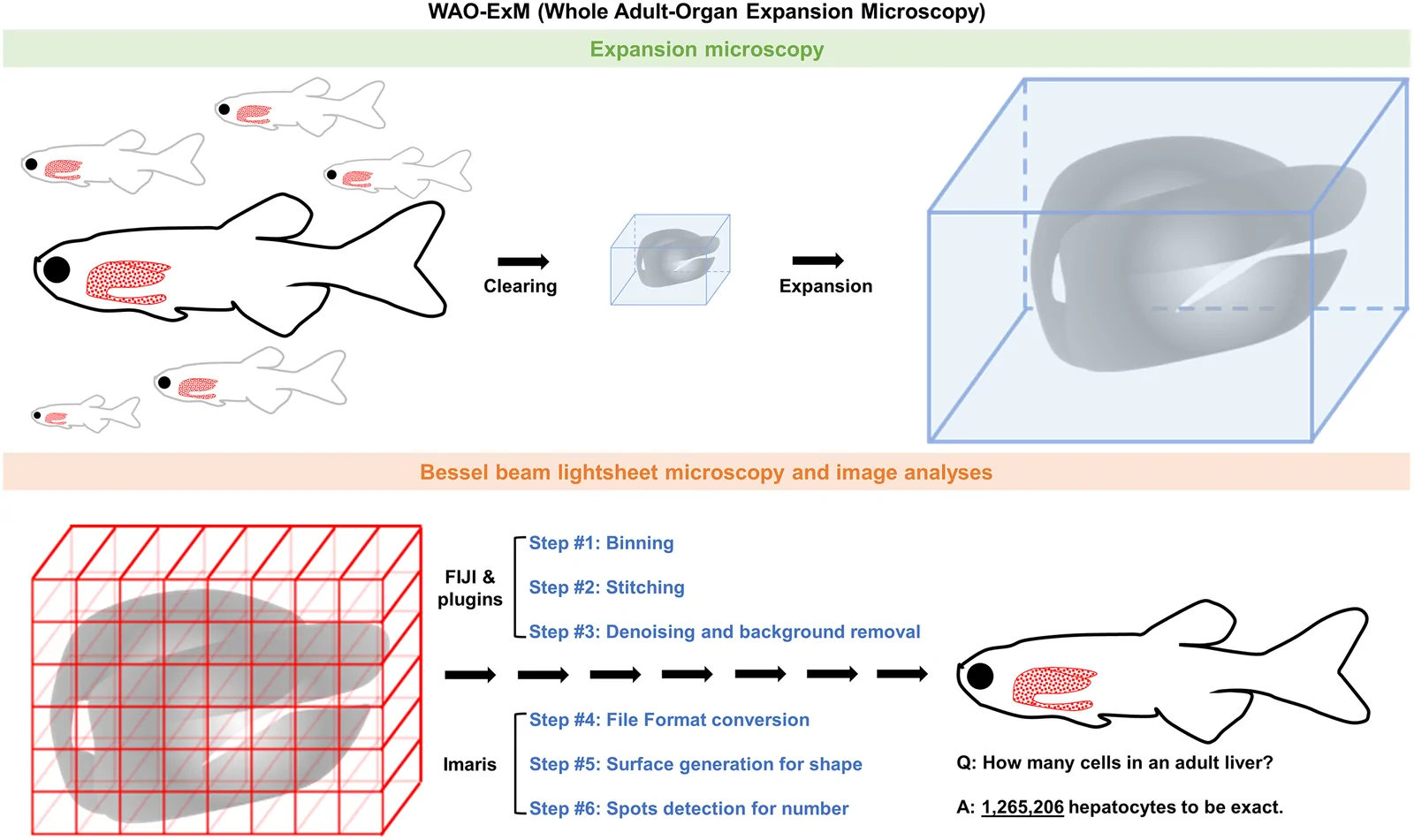
WAO-ExM workflow for whole-organ single-cell imaging and analysis. Schematic overview of the WAO-ExM sample processing, imaging, and analysis pipeline. This platform enables in toto imaging of intact adult vertebrate organs across centimeter-scale volumes while preserving native organ architecture, single-cell resolution, and cell-type specificity, thereby linking microscopic cellular behaviors to macroscopic organ growth.

It is important to note that X-ray microtomography (micro-CT) represents another powerful in toto imaging approach for the organism-level detection of cellular features in three-dimensional space [33,34]. For instance, contrast-enhanced micro-CT has been used to visualize every brain cell nucleus and melanin-containing pigment cells in an intact zebrafish larva [34,35], providing quantitative insights into developmental defects that were not accessible in earlier studies [33]. However, because micro-CT relies on chemical staining that differentially affects X-ray attenuation levels across tissue types, it can be difficult to resolve specific cell populations or tissue types that exhibit little or no contrast upon staining. Of note, a recent advance from Ueda’s group established a CUBIC and lightsheet microscopy-based framework that generates single-cell-resolved, nucleus-centered atlases of whole adult mouse organs and the neonatal body [36]. Alternatively, organism-level visualization of cellular and subcellular features can be readily achieved by integrating physical tissue sectioning with optical imaging [37,38]. Thus, we do not propose that WAO-ExM is the only approach capable of achieving both in toto scale and single-cell resolution at centimeter-scale. However, it does have some distinct advantages. When combined with the diverse repertoire of transgenic reporter lines and genetic tools (accessible from the zebrafish research community), WAO-ExM may provide a streamlined, complementary, and versatile platform for investigating in toto cellular and subcellular phenotypes and mechanisms across complete juvenile-to-adult vertebrate developmental trajectories in a cell- and organ-type–specific manner.

Beyond imaging, our study also establishes a scalable computational framework for analyzing terabyte-scale, whole-organ datasets at single-cell resolution. By integrating open-source preprocessing tools, including Fiji for binning and large-volume stitching [39], CLIJ for GPU-accelerated high-throughput image filtering [40–42], and commercial software Imaris (Oxford Instruments) for visualization and quantification, we were able to implement a hybrid workflow that allows for efficient data processing within workstation-level environments (Fig 6; detailed pipelines and codes are provided in the Methods section). Importantly, our pipeline design features both computational scalability and practical accessibility. Thus, terabyte-sized in toto datasets can be processed in standard zebrafish laboratories without the need for advanced computing infrastructure. Nevertheless, our analysis was hindered by fundamental limitations of current Imaris file formats and workflows, including their reliance on monolithic, HDF5-based file structures that limits efficient parallelization and introduces substantial storage overhead associated with the multiple format conversions required for downstream analyses. These limitations highlight the need for new imaging data architectures. Emerging standards, such as the Open Microscopy Environment Next Generation File Format (OME-NGFF) [43], built on the Zarr specification, exhibit distributed, chunked data formats that are inherently compatible with parallel and cloud-native computing frameworks. These formats can be integrated naturally into scalable computing ecosystems, including parallel and distributed computing frameworks such as Dask [44], HPC infrastructures, workflow management systems such as Nextflow [45], Snakemake [46], and Galaxy [47], as well as emerging analysis platforms such as Fractal [48]. Together, we expect these developments to accelerate the analysis of ever-growing bioimaging datasets by enabling computational workloads to be distributed beyond local workstations and across scalable computing architectures.

Since we focused our study on the post-embryonic growth period, we identified hepatocyte proliferation mechanisms most relevant to this developmental period. Future studies will be needed to determine whether similar cellular growth strategies are deployed during liver regeneration and adult homeostasis, and how these strategies may be perturbed in the context of liver disease [49–55]. Moreover, our temporal inactivation of the ECM component *lamb1a* during the post-embryonic growth period revealed that liver morphogenesis and hepatocyte number increase are not necessarily coupled. Although this finding uncovers an unexpected role of the ECM in shaping of the adult liver, it should be noted that post-embryonic changes in liver morphology are spatiotemporally correlated with the process of intestinal bending [15]. More work is thus needed to determine whether the effects of *lamb1a* on sculpting the liver are direct or if the protein acts indirectly through its pleiotropic influences on the morphogenesis of the gut and/or other adjacent organs and tissues.

In summary, this study integrates tissue clearing, expansion microscopy, lightsheet microscopy, macro-scale optics, and a streamlined analysis pipeline to achieve volumetric dissection of adult vertebrate organs at bona fide single-cell resolution. Our identification of a temporally concentrated growth phase, the emergence of dominant clones, the decoupling of organ morphology from cell proliferation, and organ-wide-specific cellular patterns highlight intricate organogenesis mechanisms that can only be appreciated when the in toto view fully spans both centimeter- and micrometer-scale dimensions.

## Materials and methods

### Ethics statement

Zebrafish were housed and maintained in accordance with standard procedures in the Institute of Cellular and Organismic Biology zebrafish facility at Academia Sinica. The study protocol was approved by the Institutional Animal Care and Utilization Committee (IACUC) at Academia Sinica (Protocol No. 20-12-1598 and 24-04-2158).

### Zebrafish

*Tg(fabp1a:Brainbow 1.0L)as79* was generated using a transgenic construct comprising the 2.8 kb *fabp1a* (also known as *lfabp*) promoter [19] and the Brainbow 1.0L sequence [21]. *Tg(fabp1a:TET3G, TETRECreERT2)as80* was created with a transgenic construct containing the *fabp1a* promoter driving the Tet3G-driven CreERT2 sequence [22]. For *Tg(fabp1a:TET3G, TETRECreERT2)*, also referred to as *Tg(fabp1a:iCre)*, a *cmlc2:nBFP2* cassette flanked by I-SceI sites was co-injected as a selection marker. *Tg(fabp1a:palm-mTurquoise; fabp1a:H2A-mCherry)as81* was generated for hepatocyte quantification. *Tg(ins:H2A-EGFP)*as82 was generated using a transgenic construct comprising the 995 bp *ins* promoter [56]. All constructs were flanked by I-SceI sites to facilitate transgenesis. The *lamb1a*^*sde1*^ temperature-sensitive mutant line (pd110) and *Tg(tcf21:nuceGFP)pd41* and *Tg(fabp10a:tert; p53−/−)* were described previously [28,29,57]. Zebrafish larvae were fed paramecia starting at 8 dpf. Animal density was maintained at 20 fish per 3 L unless otherwise specified. For Lamb1a inactivation experiments, *sde1* and *sde1*/+ animals were transferred from 25 to 34°C for the indicated durations. Of note, adult male zebrafish were consistently shown in Figs 1C, 2D, 3E, 3F, 5E, S1B, and S5A, except in S3C Fig, which included both males and females.

### Cre activation in *liverbow*

Cre activity was transiently induced in 4 dpf embryos using both doxycycline (20 μg/ml; Sigma-Aldrich, D9891) and tamoxifen (2 μM; Sigma, T5648) in all experiments. Induction was performed for 24 h, except for Fig 3G, where a 4-h induction time was used.

### Imaging and confocal microscopy

Fish were sedated with tricaine (0.4 mg/ml), and dissected livers were mounted in 1.5% low melting agarose prior to imaging. For cell heterogeneity and cell volume quantification, images were captured using a Leica SP8 upright confocal microscope with a 25× water dipping lens (25×/0.95 HCXIRAPO). For imaging *liverbow*, excitation was performed using 448 and 514 nm laser lines. Emission was collected with the following bandpass filters: (1) 450–510 nm bandpass (CFP channel), (2) 520–550 nm bandpass (GFP channel), and (3) 605–650 nm bandpass (RFP channel). For imaging *Tg(fabp1a:palm-mTurquoise; fabp1a:H2A-mCherry)*, 448 and 552 nm lasers were used for excitation. Emission was collected with bandpass filters set to 455–520 nm bandpass (CFP channel) and 595–650 nm bandpass (RFP channel). Bright field images were captured using an epifluorescence microscope (Leica M205) equipped with a highly sensitive sCMOS camera (Leica DFC9000 T). For histology, images were captured using SP8 confocal with a 20× lens (Leica SP8; HC PL APO 20×/0.75 CS2, WD 0.62 mm).

### RT-qPCR

Liver tissues were collected from 4 individuals under a dissecting microscope, and each tissue was homogenized in 1 ml Trizol (Sigma, Cat. No: T9424-200ML) using a TissueLyser II (Qiagen). cDNA was synthesized from 0.6 μg of total RNA using the SuperScript III First-Strand Synthesis System (Invitrogen, Cat. No: 18080051). qPCR analysis was performed with a Roche LightCycler 480 following the manufacturer’s instructions. Primer sequences are listed in S1 Table. Each condition included four biological replicates and three technical replicates. Results were analyzed using the *ΔΔC*_*T*_ method, with *rpl13a* expression serving as the internal control.

### Sample processing and expansion microscopy

#### Whole liver tissue processing.

The immunostaining procedure for mCherry was adapted from previous studies [58,59]. In brief, freshly collected 7 and 21 dpf fish were fixed in 4% paraformaldehyde (PFA) supplemented with 0.2% glutaraldehyde (GA) for 3 days at 4 °C, followed by three washes in 1× PBS (15 min each). Liver-containing trunk tissues were collected for further processing. Samples were blocked in blocking buffer (10% normal goat serum [NGS], 2% Triton X-100, 0.02% sodium azide in 1× PBS) at 37 °C for 2 h prior to primary antibody incubation (anti-mCherry [GT857], GTX630189; 1:100 dilution) in antibody dilution buffer (1% NGS, 0.25% Triton X-100, 0.02% sodium azide in 1× PBS) for 3 days at room temperature with gentle shaking. Samples were washed three times in wash buffer (1% Triton X-100 in 1× PBS; 1 h each) and subsequently incubated with secondary antibody (Alexa Fluor 568–conjugated goat anti-mouse IgG [H + L], cross-adsorbed; 1:100, A-11004) for 1 day at room temperature. After three additional washes (1 h each), samples were post-fixed in 4% PFA supplemented with 0.2% GA for 2 h, washed in PBS, and processed for 10× -KA-ExM.

For 42 dpf and adult samples, livers were dissected and fixed in 4% PFA for 3 days at 4 °C. Fixed tissues were subjected to delipidation using a combination of 1% heptakis (2,6-di-O-methyl)-β-cyclodextrin and 1× CUBIC-L at 37 °C. Samples were then blocked in blocking buffer (10% NGS, 2% Triton X-100, 0.02% sodium azide in 1× PBS) for 2 h at 37 °C with gentle shaking. Antibodies were diluted in immunostaining buffer (5 mM HEPES, pH 7.5, 5% Triton X-100, 100 mM NaCl, 0.25% casein, 2.5% Quadrol, 0.5 M urea) [59], and samples were incubated with primary antibody (anti-mCherry [GT857], GTX630189; 10 µL per 1 mL buffer per sample) for 5 days at room temperature with gentle shaking. Samples were washed three times in wash buffer (0.1 M PBT; 1 h each) and incubated with secondary antibody (Alexa Fluor 568–conjugated goat anti-mouse IgG [H + L], 1:100) for 3 days at room temperature. After three washes in wash buffer (1 h each) and two additional washes in 1× PBS (15 min each), samples were post-fixed in 1% formaldehyde overnight at room temperature with gentle shaking. Finally, samples were washed three times in 1× PBS (5 min each) prior to 4× -KA-ExM processing.

#### Tissue clearing (no expansion).

CUBIC clearing of adult liver samples was performed as previously described with minor modifications [58]. Following mCherry immunostaining, samples were subjected to delipidation in CUBIC-L buffer at 37 °C for 3 days with daily buffer exchange. After delipidation, samples were washed in 1× PBS for 1 day with at least five buffer changes to ensure complete removal of clearing reagents. Samples were then immersed in CUBIC-R+ for refractive index (RI) matching at room temperature for 2 days with gentle shaking. The RI matching solution was refreshed once prior to imaging.

#### Tissue clearing (with expansion; 10× -KA-ExM and 4× -KA-ExM).

The procedure for 10× -KA-ExM were performed as previously described [2]. In brief, for KA-ExM of 7 and 21 dpf liver samples, tissues were first anchored using methacrylic acid N-hydroxysuccinimide ester (MA-NHS; 10 mM) overnight at 4°C, followed by two washes in 1× PBS (5 min each) prior to gelation. For gel preparation, one tube of KA monomer stock (450 µL) was subjected to degassing by vacuum for 10 min, followed by N_2_ gas equilibration for 10 min; this cycle was repeated once. Immediately before use, 50 µL of potassium peroxydisulfate (KPS; 0.036 g/mL in ddH_2_O) and 2 µL of 100% tetramethylethylenediamine (TEMED) were added to the degassed monomer solution. The resulting gelling solution (100 µL) was dispensed onto a parafilm-lined 30-mm dish, and a 15-mm coverslip bearing the sample was placed cell-side down onto the droplet. Gelation was carried out at 37 °C for 2 h in a humidified chamber. Following gelation, coverslips were removed, excess gel was trimmed, and samples were transferred to digestion buffer (50 mM Tris, pH 8.0; 800 mM guanidine HCl; 2 mM CaCl_2_; 0.5% Triton X-100 in ddH_2_O) supplemented with freshly added proteinase K (1:100 dilution). Digestion was performed overnight at room temperature with gentle shaking. The following day, gels were washed three times in 1× PBS (15 min each) and stained with DAPI (1:3,000 in PBS; ThermoFisher 62248) for 4 h at room temperature. Samples were then washed three times in PBS (5 min each) and expanded in ddH_2_O through three sequential incubations (30 min each), followed by overnight expansion at room temperature. Fully expanded samples were mounted for lightsheet imaging the next day.

For 4× -KA-ExM of 42 dpf and adult liver samples, immune-stained tissues were pre-incubated in 4× -KA-ExM gelling solution for 2 days at 4 °C, as previously described [9,60]. Fresh gelling solution was prepared by combining monomer stock with 1% (w/v) VA-044 initiator. Samples were placed between two 15-mm coverslips in a gelation chamber, and 200 µL of gelling solution was added per sample. Gelation was performed at 37 °C for at least 2 h in a hybridization oven until complete polymerization. Excess gel was trimmed, and samples were subjected to denaturation in SDS buffer (200 mM SDS, 20 mM Tris) at 70 °C for 2 days with gentle shaking. Following denaturation, samples were washed three times in 1× PBST (PBS with 0.2% Triton X-100) at 37 °C for 2 h each, followed by two washes in 1× PBS at room temperature (30 min each). After additional trimming, samples were stained with DAPI (1:3000 in PBS) for 1 day at room temperature. DAPI-stained samples were briefly washed in PBS and expanded in ddH_2_O with three water exchanges (30 min each) on a shaker. Fully expanded samples were maintained in ddH_2_O at room temperature until lightsheet imaging.

#### Liver ventral lobe processing (42 dpf).

The immunostaining of GFP (anti-GFP, Rabbit/Polyclonal Antibody, ThermoFisher A11122) for 42 dpf liver ventral lobe was the same as mCherry staining for 42 dpf whole liver as described above, except the incubation time for primary antibody was shortened to 3 days, and the incubation for the secondary antibody was shortened to 1 day [Goat anti-Rabbit IgG (H + L) Cross-Adsorbed Secondary Antibody, Alexa Fluor 488, ThermoFisher A11008]. The expansion process was the same as described for 42 dpf whole liver samples.

#### Lightsheet microscopy.

The entire 4× -KA-expanded 42 dpf and adult liver samples were imaged using a custom-built scanning beam light-sheet microscope provided by the Light Sheet Microscopy Core Facility at Academia Sinica. Samples were mounted on a 3D-printed imaging holder as previously described [9,60]. The camera exposure time was set to 85 ms, and laser illumination was controlled by an Arduino-based system that relayed the camera “exposure all” signal to the laser control unit. A pair of Olympus 4× objectives (NA = 0.28, WD = 28.8–30.0 mm) were used for excitation, while a 10× Olympus XLPLN10XSVMP objective (NA = 0.6, WD = 8 mm) was used for detection; the detection lens was coupled with a 150 mm tube lens, resulting in a final lateral resolution of 0.78 µm/pixel. The entire 10×-KA-expanded 7 dpf and 21 dpf liver samples were imaged using a separate custom-built light-sheet system based on an axicon-generated Bessel beam configuration, equipped with two long-working-distance objectives. The excitation objective (NA = 0.5, WD = 11.7 mm) was custom-designed to enhance optical sectioning, while an extra-long-working-distance detection objective (Olympus, NA = 0.7, WD = 8 mm) enabled signal collection from centimeter-scale depths. Tilt scanning was performed along the x- and y-axes using a voice-coil stage (V308, Physik Instrumente) with up to 7 mm travel range along the z-axis. Of note, no tilt scanning along the z-axis was required with this setup.

The 4×-KA-expanded 42 dpf ventral lobe samples were imaged using a Zeiss Light-sheet Z.1 system. Image acquisition was performed at 1,920 × 1,920 pixels, 16-bit depth, with 0.5-1× zoom, using 10×/0.2 illumination objectives and a W Plan-Apochromat 20×/1.0 detection objective. Excitation wavelengths of 405 nm (laser power 3%) and 488 nm (laser power 1%) were used for imaging DAPI-labeled nuclei and GFP signals, respectively. Two separate tracks were used for each channel, with frame-fast switching enabled. The system was configured with a laser blocker (LBF 405/488/561/640) and emission filters (SBS LP560 combined with BP 505–545 nm). The exposure time was set to 99.9 ms, and the light-sheet thickness was 3.07 µm. Z-stack images were acquired in continuous drive mode with a z-step size of 5 µm.

### Image processing and analysis

#### Preprocessing.

To enable processing of the large-volume raw image datasets generated by lightsheet microscopy on a single workstation, raw images acquired from the custom-built lightsheet microscope were first spatially binned by a factor of 4 in the x–y dimensions. For datasets exhibiting pronounced illumination bias, flat-field correction was applied before stitching using the BaSiC plugin in Fiji [61]. Tiled images were then stitched to reconstruct the complete volumetric datasets. Images acquired using the custom-built lightsheet microscope were stitched with the Grid/Collection stitching plugin in Fiji [62], whereas those acquired using the Zeiss Lightsheet Z.1 were stitched with arivis Vision4D ZEISS Edition. To enhance nuclei detection, slice-by-slice filtering was applied. A Difference of Gaussians (DoG) filter was used for datasets acquired with the custom-built lightsheet microscope, whereas a Top-Hat filter was used for those acquired with the Zeiss Lightsheet Z.1. For anatomical segmentation of the liver based on hepatocyte-mCherry signal, a Gaussian blur filter was applied to smooth tissue boundaries and improve subsequent liver surface extraction. These preprocessing steps were implemented in Fiji [39] using custom ImageJ macros (source code: https://github.com/WeiChenChu/LLSM-Batch-Preprocessing), and executed with GPU acceleration via the CLIJ2 framework [40–42]. Following preprocessing, datasets were converted into the Imaris-compatible (.ims) format using ImarisFileConverter (version 9.9.1; Oxford Instruments) for subsequent visualization and quantitative analysis in Imaris (version 9.9.1; Oxford Instruments). Movies were rendered in Amira using custom Python scripts for spot detection and image processing.

#### Cell number quantification.

Liver cell numbers were quantified using the Spots Detection module in Imaris. To ensure anatomical accuracy, a three-dimensional liver surface was generated from the Gaussian-blurred hepatocyte-mCherry signal using the Surfaces module to define the region of interest (ROI). Detected spots from both the DAPI and mCherry channels were then spatially filtered against this surface to automatically exclude signals located outside the hepatic boundary. Cell numbers in the pancreas were quantified directly from the stitched raw image datasets using the Spots module in Imaris without additional preprocessing or spatial filtering. Of note, the same detection settings in the Imaris Spots module were applied across datasets acquired using the same microscope and acquisition settings. Spatial parameters were defined in calibrated physical units (µm) based on the observed spot size in the expanded images, without further correction for the expansion factor. Finally, all results were manually inspected to remove false-positive spots arising from residual noise, imaging artifacts, or non-specific signals. To quantify nuclei within GFP-positive clones in dissected liver tissue, a three-dimensional surface was first generated from the GFP signal using the Surfaces module in Imaris. Nuclei were then detected using the Spots module on the DAPI channel without preprocessing filters. To restrict quantification specifically to the GFP-positive clone, detected DAPI spots were spatially filtered against the GFP-derived surface, thereby excluding nuclei outside the defined region of interest.

### Statistical analysis

Statistical tests were performed with Prism 9.0 (GraphPad Software). All statistical values were displayed as mean ± standard deviation (s.d.). A two-tailed Student *t* test for parametric distributions was used when distributions passed the D’Agostino–Pearson normality test. Otherwise, a two-tailed Mann–Whitney test was used for non-parametric distributions. Sample sizes and statistically significant differences are reported in the corresponding ﬁgure legends.

## Supporting information

S1 Fig The zebrafish hepatocytes are predominantly mononucleated throughout larval and adult stages. **(A)** Schematic (left) and confocal image (right) of a 7 dpf double transgenic fish *Tg(fabp1a:palm-mTurquoise; fabp1a:H2A-mCherry)*, labeling hepatocyte membranes (blue) and nuclei (red). **(B)** Representative confocal optical sections showing hepatocyte membrane (gray) and nuclei (red) at 7 dpf, 21 dpf, 42 dpf, and adult stages. The yellow arrow and zoomed-in inset highlight a binucleated hepatocyte. Scale bar, 10 µm. **(C)** Quantification of the percentages of mononucleated and multinucleated hepatocytes across developmental stages. The numbers of hepatocytes identified in each category are indicated, and annotated values in the pie charts denote the percentage of mononucleated hepatocytes. The numbers of analyzed fish and total cells are noted below. **(D)** Quantification of hepatocyte volumes across developmental stages. Data are shown as mean ± s.d.; the number of fish examined is indicated, and the annotated cell numbers indicate the total cell number of hepatocytes analyzed. Source data are available in S2 Data.
(TIF)

S2 Fig Potassium acrylate-based hydrogels readily expand intact zebrafish larvae by 10-fold. **(A)** Whole-mount epifluorescence images of 7 dpf larvae before (1×) and after (10×) expansion. Scale bar, 1 mm. **(B)** WAO-ExM images of a 7 dpf liver before (NE, non-expanded) and after (WOA-ExM) expansion (stitched). Scale bar, 200 µm. **(C)** High-magnification comparison of nuclear signals before (NE, non-expanded) and after WAO-ExM expansion. Purple lines denote the region used for signal intensity analysis, and asterisks mark heterochromatin structures. Scale bar, 20 µm. The accompanying quantification shows the improved z-axis signal-to-background ratio following expansion. Source data are available in S2 Data.
(TIF)

S3 Fig Dominant hepatocyte clones occupy spatially distinct positions in the ventral lobe. **(A, B)** Confocal images of livers at 42 dpf and adult stages in the absence of doxycycline and tamoxifen treatment (stitched). Of note, no leaky recombination was detected in any of the 13 and 18 individuals examined at the respective time points. White dashed line delineates the ventral lobe boundary. Liver lobes are indicated by color-coded labels: left (L, yellow), right (R, green), and ventral (V, magenta). Scale bar, 1 mm. **(C)** Representative examples of dominant hepatocyte clones captured in adult livers (stitched). White dashed lines mark the ventral lobe boundaries. Scale bar, 1 mm.
(TIF)

S4 Fig Lamb1a inactivation readily compromises liver morphogenesis during post-embryonic growth. **(A)** RT-qPCR analysis of *lamb1a*, *lamc1*, *lef1*, and *ddx52* transcript levels in livers from 28 dpf and adult (3 mpf) stages. Data are shown as mean ± s.e.m. (*n* = 4 biological replicates). Significance was examined by two-tailed Student *t* test. **(B)** Pre-expansion whole-mount epifluorescence images showing liver morphology in control (*sde1/+*) and mutant (*sde1)* animals. Of note, these four mutant fish were subsequently processed for WAO-ExM, with the corresponding hepatocyte counts reported in Fig 4E and 4F. Asterisk marks intestinal autofluorescence. Scale bar, 1 mm. Source data are available in S2 Data.
(TIF)

S5 Fig In toto visualization of all pancreatic beta cells in adult zebrafish. **(A)** Schematic and bright-field overview of an adult pancreas prior to expansion (NE, non-expanded). Orange dashed lines outline the pancreas, green fluorescence marks beta cells, and the red asterisk indicates the primary islet processed using WAO-ExM. Scale bar, 1 mm. **(B)** WAO-ExM images of a 4× expanded adult pancreas (left; stitched), and a high-magnification view of the image showing nuclear heterochromatin structures (right). Scale bars, 250 µm. **(C)** Quantification of total beta cell numbers in the adult pancreas. Data are shown as mean ± s.d.; the number of fish examined is indicated below the x-axis, and the annotated cell numbers represent mean values. Source data are available in S2 Data.
(TIF)

S1 Movie Whole adult-organ expansion microscopy (WAO-ExM) image of a 4× expanded adult liver. A total of 6 × 15 tiles and 2,443 z-steps were acquired, covering a three-dimensional volume of 9.6 × 21.2 × 5.3 mm^3^ in the expanded sample space.
(MP4)

S2 Movie Three-dimensional light-sheet microscopy image of a non-expanded adult liver after tissue clearing. A total of 4 × 5 tiles and 3,764 z-steps were acquired, covering a three-dimensional volume of 6.2 × 7.6 × 1.6 mm^3^ in the sample space.
(MP4)

S3 Movie WAO-ExM image of a 10× expanded 7 dpf larval liver. A total of 2 × 2 tiles and 147 z-steps were acquired, covering a three-dimensional volume of 0.54 × 0.54 ×× 0.54 mm^3^ in the sample space. Of note, the 10× expansion readily enables subcellular, submicrometer-resolution imaging of nuclear chromatin architecture at the single-cell level.
(MP4)

S4 Movie Lightsheet microscopy image of a non-expanded 7 dpf liver after tissue clearing. A total of 7 × 7 tiles and 349 z-steps were acquired, covering a three-dimensional volume of 2.8 × 2.8 × 2.0 mm^3^ in the sample space.
(MP4)

S5 Movie WAO-ExM image of a ventral liver lobe expanded 4× at the 42 dpf juvenile stage. In *Tg(fabp1a:brainbow; fabp1a:iCre*) animals, all hepatocytes are labeled by default, and a single hepatocyte-derived clone is shown in blue. The clone volume was overlaid with DAPI nuclear signals to estimate the total number of cells within the clonal domain.
(MP4)

S6 Movie Whole-liver morphologies of control (*sde1/+*) and *lamb1a*-affected (*sde1*) fish. Three-dimensional surface reconstructions were generated from WAO-ExM images using the Imaris surface-rendering function.
(MP4)

S7 Movie WAO-ExM image of a 4× expanded adult heart, showing *Tg(tcf21:nuceGFP)*-labeled epicardial cells. A total of 4 × 4 tiles and 298 z-steps were acquired, covering a three-dimensional volume of 6.4 × 6.4 × 3.0 mm^3^ in the sample space.
(MP4)

S8 Movie WAO-ExM images of a 4× expanded adult pancreas, showing *Tg(ins:H2A-EGFP)*-labeled beta cells. A total of 2 × 4 tiles and 128 z-steps were acquired, covering a three-dimensional volume of 1.7 × 1.7 × 0.9 mm^3^ in the sample space.
(MP4)

S1 Data Source data for Figs 1–5. An Excel spreadsheet containing the underlying numerical data for Figs 1E, 2B, 2E, 3I, 4C, 4F, 5B, 5D, and 5J.
(XLSX)

S2 Data Source data for S1–S5 Figs. An Excel spreadsheet containing the underlying numerical data for S1C, S1D, S2C, S4A, and S5C Figs.
(XLSX)

S1 Table List of RT-qPCR primers. (XLSX)

## Abbreviations

DoG: Difference of Gaussians
dpf: days post-fertilization
ECM: The extracellular matrix
ExM: expansion microscopy
IACUC: Institutional Animal Care and Utilization Committee
NE: unexpanded liver
NGS: normal goat serum
RI: refractive index
ROI: region of interest
WT: wild-type
