# Supplementary material for: Whole-organ single-cell mapping defines growth dynamics and clonal organization in developing and adult zebrafish livers
Source: PLoS Biol. 2026 Sep 28;24(9):e3004007. doi: 10.1371/journal.pbio.3004007 (PMC13641717; doi:10.1371/journal.pbio.3004007)
Supplement: Supplementary file 1 [file pbio.3004007.s019.pdf]

Point-by-point responses

Academic Editor: "you should soften the claim of a new method because imaging of larger tissue (like the brain) has already been done, and citing those additional papers that performed larger tissue imaging would be helpful".

Response (1): We very much appreciate the Editor's comments. We have revised the following text to more precisely define the advances of our study and acknowledge additional pioneering studies that used expansion microscopy-based large-scale tissue imaging.

On page 2 of the Abstract, we now describe WAO-ExM as "an integrated workflow" rather than as a method:

> "Here we establish an integrated workflow of whole adult-organ expansion microscopy (WAO-ExM) that enables in toto single-cell-resolved visualization of every hepatocyte within a complete adult vertebrate liver."

On page 4, paragraph 1, we highlight key studies that advanced the technology to enable centimeter-scale imaging of the postnatal mouse brain:

> "For instance, this combined approach can readily enable optical imaging of individual cells and neurons across the entire expanded, centimeter-scale postnatal mouse brain (up to 5 x 2.4 x 3.7 cm^3) at an effective resolution of ~300 nm [6, 9-11]"

Reviewer #1: Sumeet Pal Singh – note that this reviewer has signed the review.

Roan et al. present the development and application of whole adult-organ expansion microscopy (WAO-ExM) for single-cell-resolved imaging of intact zebrafish organs. The authors combine tissue clearing, expansion microscopy, light-sheet imaging and large-volume image analysis to quantify hepatocyte numbers across post-embryonic liver growth. They further integrate this approach with clonal lineage tracing, genetic perturbation of laminin function and analyses of additional organs.

The principal strength of the study is the exceptionally high-quality imaging. The whole-organ reconstructions are visually impressive and provide a clear demonstration of the technical capabilities of WAO-ExM. In particular, the preservation of cellular resolution across millimetre-scale samples and through considerable tissue depth is convincing. The images of the developing and adult liver, the multicolour clonal analysis and the extension of the method to the heart and pancreas are of excellent quality. The accompanying image-processing workflow should also be valuable to laboratories working with large volumetric datasets.

The biological observations are interesting. The quantitative analysis identifies a pronounced increase in hepatocyte number between 21 and 42 dpf and confirms that post-embryonic liver growth is associated with substantial heterogeneity in clonal contribution. The finding that individual clones

can occupy a large fraction of the ventral lobe is particularly striking. The laminin experiments further suggest that changes in tissue morphology can occur without a comparable change in total hepatocyte number.

Overall, this is a technically strong and carefully performed study that should be of broad interest to researchers working in developmental biology, organ growth and quantitative imaging. The manuscript is generally well written, although several conclusions would benefit from more cautious interpretation and some additional methodological clarification.

Major comments

1. Interpretation of the developmental growth burst

The large increase in hepatocyte number between 21 and 42 dpf is an important observation. However, the present data consist of relatively widely separated developmental time points. The conclusion that liver growth occurs through a sharply defined proliferative burst may therefore be somewhat stronger than the temporal resolution permits. The authors could either moderate this wording or, where feasible, include one or two intermediate time points. Or include quantification of cell proliferation using EdU or Ki67 / PCNA.

Even without additional experiments, it would be appropriate to describe this interval as a period of markedly accelerated hepatocyte accumulation rather than implying that the precise timing and duration of the proliferative phase have been fully resolved.

Response (2.1): We are very thankful to the reviewer for his enthusiastic comments and careful reading. We agree that the temporal resolution of our current dataset does not allow us to precisely define the onset or duration of the proliferative burst. We have now acknowledged this limitation on page 9, paragraph 1:

> "Because our measurements were obtained at discrete developmental time points, the precise onset, and duration of this accelerated growth remain to be determined through finer temporal sampling. Nonetheless, by defining the absolute number of hepatocytes in a growing individual, we demonstrated that liver growth is not gradual but instead accelerates markedly within this three-week developmental window."

2. Quantification and interpretation of dominant clones

The clonal analysis is visually compelling and is one of the strongest parts of the manuscript. However, the estimate of cell number within each labelled clone is based on DAPI-positive nuclei contained within a GFP-defined volume. As the authors acknowledge, this measurement may include endothelial, immune, biliary and other non-hepatocyte nuclei.

The cell-number estimates should therefore be described more explicitly as the number of nuclei contained within the clonal territory rather than the exact number of hepatocytes in the clone. Alternatively, the authors could restrict the analysis to nuclei positive for a hepatocyte-specific

nuclear marker. This distinction is important because the estimated clone sizes are used to support the conclusion that individual hepatocytes generate more than 10,000 descendants.

Response (2.2): Because our clonal analysis relies on the *liverbow* reporter fish, which is incompatible with our hepatocyte nuclear reporter (i.e., *fabp1a:H2A-mCherry*) as both encode red fluorescent proteins, we thus used DAPI-positive nuclei within the clonal territory as a proxy for the cell number. We have now explicitly pointed out this limitation on page 10, paragraph 1:

> "Of note, the calculated cell number does not represent an exact count of hepatocyte descendants in this context, and is likely an overestimate, as nuclei from interstitial and perivascular cells may also be included within the quantified domain."

The manuscript should also clarify how confidently a large, uniformly coloured territory can be assigned to a single original hepatocyte. Although sparse induction substantially reduces the likelihood of adjacent cells independently acquiring similar colours, Brainbow reporters do not necessarily provide a unique barcode for every labelled cell. The possibility of similarly labelled neighbouring clones should be addressed.

Response (2.3): We thank the reviewer for raising this important point. Although we showed that both standard and sparsely induced labeling conditions generated dominant clones of similar sizes (Fig 3D-3I and S3C Fig), and we found dominant clones in 34 out of the 35 adult livers examined, we cannot completely exclude the possibility that some color territories may have originated from two adjacent hepatocytes that independently acquired similar colors. We have now included the total number of adult animals examined in the text and in Fig 3E, which shows representative clonal profiles, and have acknowledged this limitation.

On page 9, paragraph 2:

> "Remarkably, the emergence of dominant hepatocyte clones in both juvenile and adult livers was highly evident, particularly in the ventral lobe and distal tip regions (Fig 3D-3F). These clones were readily captured in 34 of the 35 adult livers examined, with representative images shown in Fig 3E, 3F, and S3C Fig. These findings were in line with previous lineage-tracing studies of embryonic hepatocyte progenitors [15]."

On page 10, paragraph 1:

> "Also, although both standard and reduced Cre activity generated dominant clones of similar sizes, we cannot completely exclude the possibility that some color-labeled territories comprise neighboring clones of similar colors. Nevertheless, our ability to take a whole-organ, three-dimensional view of individual hepatocyte growth profiles allowed us to conclude that like embryonic hepatocyte progenitors, postembryonic hepatocytes are not equally proliferative. Instead, a subset of cells selectively and dramatically expands in a spatially restrictive manner, likely accounting for the sharp burst in hepatocyte number over a short time period."

3. Laminin phenotype and intestinal morphogenesis

The lamb1a experiment provides an interesting example of organ deformation without a major change in total hepatocyte number. However, the data do not yet establish that laminin acts directly within the liver to determine liver shape. Lamb1a has broad developmental functions, and the temperature-sensitive perturbation is systemic.

Importantly, the post-embryonic remodelling of zebrafish liver morphology occurs in close physical association with intestinal bending and folding. The abnormal liver shapes observed after lamb1a inactivation could therefore arise secondarily from altered intestinal morphogenesis, changes in intestinal folding or altered mechanical constraints imposed by surrounding tissues. The authors acknowledge this possibility in the Discussion, but it should be given greater prominence when interpreting the results.

Accordingly, statements such as "laminins regulate liver growth by defining tissue shape" or that liver shape and hepatocyte proliferation are independently regulated should be moderated. The present data show that systemic lamb1a inactivation alters liver morphology without measurably changing total hepatocyte number. Determining whether this is a liver-autonomous ECM phenotype or an indirect consequence of abnormal intestinal folding will require tissue-specific perturbation or simultaneous analysis of intestinal morphology.

Response (2.4): We fully agree with the reviewer's interpretation, and have removed the statement and revised the text on page 11, paragraph 1:

> "Although our *lamb1a* perturbation was systemic rather than liver-specific, and its effects may have been indirect, potentially resulting from altered gut morphogenesis [15], this unexpected finding suggested that laminins may primarily influence liver growth and development by modulating the overall organ shaping process rather than by controlling hepatocyte proliferation."

It would be useful to show the liver together with the adjacent intestine in control and mutant animals, where possible. Even a descriptive comparison of intestinal bending and liver positioning would help readers assess this alternative explanation.

Response (2.5): We are very thankful to the reviewer for this constructive suggestion. We agree that examining the liver together with the adjacent intestine could provide useful descriptive information. However, because the relationship between intestinal bending and liver positioning reported by Unterweger et al. (2023) is correlative rather than demonstrably causal, such a comparison alone may not allow us to distinguish between the direct and indirect effects of *lamb1a* perturbation. Also, visualizing the native spatial relationship between the liver and adjacent intestine is technically challenging without incorporating additional gut reporter lines (see S4B Fig). We have now pointed out these limitations on page 17, paragraph 1:

> "Although this finding uncovers an unexpected role of the ECM in shaping of the adult liver, it should be noted that post-embryonic changes in liver morphology are spatiotemporally correlated with the process of intestinal bending [15]. More work is thus needed to determine

whether the effects of lamb1a on sculpting the liver are direct or if the protein acts indirectly through its pleiotropic influences on the morphogenesis of the gut and/or other adjacent organs and tissues."

4. Statistical power in the lamb1a analysis

The whole-organ imaging of the lamb1a mutants is impressive, but the quantitative analysis includes six controls and four mutants. In addition, only approximately one-third of mutant animals developed severe liver abnormalities. It is not entirely clear whether the four animals used for hepatocyte counting were selected based on morphological phenotype.

The authors should clarify the selection criteria and report the individual morphological category for each quantified liver. A comparison between morphologically affected mutants, unaffected mutants and sibling controls would be more informative than grouping all mutants together. With the current sample size, the absence of a statistically significant difference should not be interpreted as proof that hepatocyte number is unchanged. Reporting an effect-size estimate and confidence interval would make the conclusion more precise.

Response (2.6): We apologize for not clearly describing the selection criteria for the four mutants analyzed in Fig 4E and 4F. All four mutants were selected because they exhibited pronounced liver morphological abnormalities in pre-expansion whole-mount epifluorescence images, as shown individually in S4B Fig. We have revised the text and figure legend to clarify the connection between Fig 4E and S4B Fig, and to report the number of animals in each morphological category.

Also, given the small sample size, we agree that the absence of a statistically significant difference should not be interpreted as definitive evidence that hepatocyte number is unchanged. We have revised the text to emphasize the limited nature of the dataset, and now also report the estimated mean difference and its 95% confidence interval as advised.

On page 11, paragraph 1:

> "Nevertheless, approximately one-third of the mutant fish developed severe liver morphological defects (n = 13 out of 38; S4B Fig), including complete loss of a major lobe (4 out of 13) or disproportionate lobe growth (9 out of 13). In contrast, no such defects were detected in any heterozygous siblings (n = 37 out of 37). Intriguingly, using WAO-ExM, we found that, despite these pronounced morphological defects, the total hepatocyte number did not differ significantly between sibling controls and deformed mutant livers in this limited dataset (195,242 versus 187,970 cells, respectively; estimated mean difference, -7,273 ± 36802 cells; 95% CI, -96,101 to 81,556; Fig 4D-4F; the four mutant fish analyzed by WAO-ExM were the same morphologically affected animals shown in S4B Fig)"

On page 39, Fig 4F:

> "(F) Quantification of total hepatocyte number in control (*sde1/+*) and mutant (*sde1*) fish. Data are shown as mean ± s.d.; the number of fish examined is indicated below the x-axis, and the

annotated cell numbers are mean values. Statistical significance was assessed using a two-tailed Mann-Whitney test. The resulting p-value is identical to that shown in Fig 4C. Of note, the four mutants analyzed by WAO-ExM here were the same morphologically affected animals shown in S4B Fig."

On page 43, S4B Fig:

"(B) Pre-expansion whole-mount epifluorescence images showing liver morphology in control (*sde1/+*) and mutant (*sde1*) animals. Of note, these four mutant fish were subsequently processed for WAO-ExM, with the corresponding hepatocyte counts reported in Fig 4E and 4F. Asterisk marks intestinal autofluorescence. Scale bar, 1 mm."

5. Analysis of the liver cancer model

The application of WAO-ExM to the cancer model demonstrates the potential utility of the method. However, this section is relatively preliminary. The reduction in total hepatocyte number is accompanied by reduced body length, and the p-value is close to the conventional significance threshold. The suggestion that the phenotype reflects competition between proliferative cancer cells and normal hepatocytes is speculative and is not directly tested.

The authors should substantially moderate this interpretation. Reduced hepatocyte number could reflect developmental delay, altered differentiation, cell death, changes in reporter expression or broader effects of the transgenic and p53-mutant background. The section would be stronger if it were presented primarily as a proof-of-principle application of WAO-ExM to diseased tissue.

The manuscript should also clarify whether the fabp1a:H2A-mCherry reporter remains uniformly expressed in transformed hepatocytes. If reporter expression is reduced or heterogeneous in the cancer model (due to dedifferentiation), hepatocyte counting could underestimate the total cell population.

Response (2.7): We agree with the reviewer that the findings from the cancer model remain preliminary and require more cautious interpretation, as we cannot distinguish normal hepatocytes from transformed hepatocytes in this model. As advised, we have substantially moderated this section, and explicitly pointed out the limitations of our analysis, and included alternative explanations for the findings.

On page 12, paragraph 2:

"We first noted that, at 21 dpf, the transgenic fish exhibited signs of developmental delay, as indicated by the bending angle of the posterior notochord [1] (Fig 5A) and a moderate reduction in Standard Length (~16%; Fig 5B). Intriguingly, WAO-ExM revealed that these previously established cellular-level histological abnormalities were accompanied by a 66% lower number of reporter-positive hepatocyte nuclei in transgenic animals than in age-matched wild-type controls (23,049 vs 7,769; n = 3 and 4, respectively; Fig 5C and 5D). Although preliminary and seemingly counterintuitive, given that cancerous tissues are often associated with increased cell

proliferation, these findings demonstrate the potential of WAO-ExM to quantify organ-wide cellular alterations in diseased tissue. Meanwhile, it is important to note that the lower number of reporter-positive hepatocyte nuclei could reflect developmental delay, altered hepatocyte differentiation, cell death, or reduced or heterogeneous expression of the *fabp1a:H2A-mCherry* reporter in transformed hepatocytes. Further experiments will be required to distinguish among these possibilities."

Minor comments

1. The manuscript alternates between fabp10a and lfabp when describing the reporter. The nomenclature should be standardised.

Response (2.8): *fabp1a* and *lfabp* refer to the same gene in the literature. We have now reviewed and standardized the nomenclature throughout the manuscript and added a footnote to clarify this on page 18, paragraph 1:

> "*Tg(fabp1a:Brainbow 1.0L)as79* was generated using a transgenic construct comprising the 2.8 kb *fabp1a* (also known as *lfabp*) promoter [19] and the Brainbow 1.0L sequence [21]."

2. The statement that adult zebrafish liver contains an average of 1,265,206 "cells" should specify "hepatocytes," since the liver also contains several non-parenchymal cell types.

Response (2.9): We have replaced "cells" with "hepatocytes" in the Abstract.

3. Sex should be reported where known, particularly for adult liver measurements. Sexual dimorphism and reproductive status may influence liver size and hepatocyte number.

Response (2.10): As advised, we have now included information on the sex of the animals in the Materials and Methods section.

On page 18, paragraph 1:

> "Of note, adult male zebrafish were consistently shown in Fig 1C, 2D, 3E, 3F, 5E, S1B, and S5A Fig, except in S3C Fig, which included both males and females."

4. Figure 1 contains visible tile-to-tile intensity variation. This does not undermine the cellular resolution, but the authors could comment on whether intensity normalisation or illumination correction was applied before segmentation.

Response (2.11): As the reviewer pointed out, the visible tile-to-tile intensity variation does not affect cellular resolution. To enable reliable segmentation across all datasets, we applied slice-by-slice filtering rather than additional global intensity normalization. As advised, we have revised the text to explicitly describe the preprocessing, stitching, and filtering steps performed before segmentation.

On page 25, paragraph 2:

> "For datasets exhibiting pronounced illumination bias, flat-field correction was applied before stitching using the BaSiC plugin in Fiji [61]. Tiled images were then stitched to reconstruct the

complete volumetric datasets. Images acquired using the custom-built lightsheet microscope were stitched with the Grid/Collection stitching plugin in Fiji [62], whereas those acquired using the Zeiss Lightsheet Z.1 were stitched with arivis Vision4D ZEISS Edition. To enhance nuclei detection, slice-by-slice filtering was applied. A Difference of Gaussians (DoG) filter was used for datasets acquired with the custom-built lightsheet microscope, whereas a Top-Hat filter was used for those acquired with the Zeiss Lightsheet Z.1."

5. The apparent reuse or inversion of labels for the supplementary movies should be checked carefully. The Results and figure legends should be cross-checked against the uploaded supplementary files.

Response (2.12): We have double-checked all labels in the supplementary movies, and confirmed their accuracy.

6. In the Methods, the authors state that identical detection parameters were used across all datasets. Given the different expansion factors and acquisition systems, it would be helpful to specify whether "identical" refers to physical dimensions after correction for expansion or to raw pixel-based parameters.

Response (2.13): We have removed the word "identical" to avoid confusion, and revised the text to specify the detection settings used.

On page 26, paragraph 2:

> "Of note, the same detection settings in the Imaris Spots module were applied across datasets acquired using the same microscope and acquisition settings. Spatial parameters were defined in calibrated physical units (μm) based on the observed spot size in the expanded images, without further correction for the expansion factor."

7. For the pancreas analysis, the authors quantify ins-reporter-positive nuclei as beta cells. The manuscript should briefly state whether smaller secondary islets were included or excluded from the reported count.

Response (2.14): We have revised both the text and the corresponding figure legend to clarify that only the primary islet was processed for imaging.

On page 13, paragraph 2:

> "WAO-ExM can also be applied to visualize and precisely enumerate cells within endocrine organs, such as pancreatic beta cells (3,152 ins-positive cells on average in the primary islet of an adult individual; S5A-S5C Fig)."

On page 43, S5A Fig:

> "(A) Schematic and bright-field overview of an adult pancreas prior to expansion (NE, non-expanded). Orange dashed lines outline the pancreas, green fluorescence marks beta cells, and the red asterisk indicates the primary islet processed using WAO-ExM. Scale bar, 1 mm."

8. "Heterogenicity" should be changed to "heterogeneity."

Response (2.15): The typo has been corrected.

9. "Compliment histological assays" should be changed to "complement histological assays."

Response (2.16): The typo has been corrected.

10. "Dox and Tam ware administered" should be corrected to "were administered."

Response (2.17): The typo has been corrected.

11. The Methods state that actb was used as the qPCR reference gene, whereas Table S1 lists primers for rpl13a. This discrepancy should be corrected.

Response (2.18): We thank the reviewer for the careful reading, and have corrected the mislabeled information.

*12. The manuscript refers to "Lamb1a inactivation" and, elsewhere, more broadly to "laminin inactivation." Since the experiment specifically perturbs *lamb1a*, the wording should remain gene-specific unless loss of the broader laminin complex has been demonstrated.*

Response (2.19): In a previous study, we showed that transient inactivation of *lamb1a* alone was sufficient to disrupt laminin complex deposition in the basement membrane, thereby perturbing basal cell polarity and signaling (Chen et al., 2015). To clarify this point, we have revised the text on page 11, paragraph 1:

> "Of note, *lamb1a* inactivation alone is sufficient to disrupt laminin complex deposition in the basement membrane [28]. Three weeks of *lamb1a* inactivation had no apparent effect on overall fish growth, as assessed by Standard Length, despite the presence of previously described fin degradation phenotypes [28] (Fig 4B and 4C)."

Reviewer #2: This beautiful and important study makes a significant contribution to our understanding of post-embryonic development and emergence of adult form, in examining cellular composition and arrangements in the liver of zebrafish from early larva to adult stages, using expansion microscopy and light-sheet imaging. The study also examines morphology of the heart, another important model for regenerative biology, and cancerous tissue, and assess clonal contributions to liver composition using a new "liverbow" transgenic system. It is fair to say the work raises the bar for what can be accomplished using these preparative and imaging modalities and will almost surely stimulate new work to better understand these kinds of phenomena across a wider range of tissues and organ systems, as well as organisms. I have only a few comments:

1. Review copy lacked Movie S1. Probably fine but check for errors in call outs etc.

Response (3.1): Because of its large file size, Movie S1 was originally provided through a separate link in the appendix. We have now reduced the file size to less than 100 MB to resolve this issue.

2. Authors use chronological age as their metric for describing developmental progress but the relationship between age and stage varies considerably across labs. For this study, 21 and 42 dpf are especially important waypoints but for some labs a 42 dpf fish will really be the same stages as a 21 dpf fish. So while dpf is certainly easy to understand and can be a useful point of reference relative to how the work flow actually proceeded, the authors need to address the potential for age/developmental stage disparity and provide a rough estimate of the true fish stages they examined at their chosen time points (and genotypes). This all likely merits no more than a sentence someplace early in the results or in a figure legend, and stages corresponding to ages can be provided in just the one place, using mm Standard Length, which is highly correlated with true developmental state across a variety of traits, retaining dpf if preferred elsewhere once its true meaning has been clarified. Note this applies also to the tumor-bearing fish that are smaller at 21 dpf; in this case an indication of size alone might be insufficient as the fish could be either developmentally at an earlier state, or they could be severely growth-deficient while nevertheless attaining the same developmental state as controls (in which case other described indicators of development stage could be reported). These kinds of benchmarks will add to rigor and reproducibility for the current study, but also work derived from it, especially important now for exploiting the true potential of the authors' methods and their own enabling of quantitative analyses.

Response (3.2): We appreciate the reviewer for raising this important point. In addition to reporting Standard Length measurements for each chronological age at which we performed WAO-ExM (Fig 2B and 5B), we have now labeled Standard Length alongside chronological age in Fig 2C and 5C.

For the tumor-bearing fish, we also assessed and highlighted a developmental-stage indicator described by Parichy et al. (2009) (i.e., the bending angle of the posterior notochord; Fig 5A), which suggests a potential developmental delay in these fish. We have substantially revised this section addressing this important consideration, cited the key reference, explicitly pointed out the limitations of our analysis, and included alternative explanations for the findings.

On page 12, paragraph 2:

> "We first noted that, at 21 dpf, the transgenic fish exhibited signs of developmental delay, as indicated by the bending angle of the posterior notochord [1] (Fig 5A) and a moderate reduction in Standard Length (~16%; Fig 5B). Intriguingly, WAO-ExM revealed that these previously established cellular-level histological abnormalities were accompanied by a 66% lower number of reporter-positive hepatocyte nuclei in transgenic animals than in age-matched wild-type controls (23,049 vs 7,769; n = 3 and 4, respectively; Fig 5C and 5D). Although preliminary and seemingly counterintuitive, given that cancerous tissues are often associated with increased cell proliferation, these findings demonstrate the potential of WAO-ExM to quantify organ-wide

cellular alterations in diseased tissue. Meanwhile, it is important to note that the lower number of reporter-positive hepatocyte nuclei could reflect developmental delay, altered hepatocyte differentiation, cell death, or reduced or heterogeneous expression of the *fabp1a:H2A-mCherry* reporter in transformed hepatocytes. Further experiments will be required to distinguish among these possibilities."

On page 39, Fig 5A:

"(A) Whole-mount epifluorescence images of 21 dpf wild-type (WT) control and cancer-prone *(fabp10a:tert; p53-/-)* fish. Red lines indicate the bending angle of the posterior notochord. Scale bar, 1 mm."

3. Results from analyzing clonal changes in composition are very intriguing. The descriptions should, however, enumerate how many individuals were examined, repeatedly, across each of the stages and how many of those were subjected to terminal expansion microscopy at the end. If most or all were single-stage analyses then the trend still seems clear but the introductory goal "to visualize each and every hepatocyte in the zebrafish liver throughout the entire post-embryonic growth period" could be misleading with respect to actual outcomes.

Response (3.3): Yes, the whole-liver view of clonal growth were single-stage analyses. As advised, we have now included the total number of adult animals examined in the text and in Fig 3E, which shows representative clonal profiles.

On page 9, paragraph 2:

"Remarkably, the emergence of dominant hepatocyte clones in both juvenile and adult livers was highly evident, particularly in the ventral lobe and distal tip regions (Fig 3D-3F). These clones were readily captured in 34 of the 35 adult livers examined, with representative images shown in Fig 3E, 3F, and S3C Fig. These findings were in line with previous lineage-tracing studies of embryonic hepatocyte progenitors [15]."

Response (3.4): The introductory goal "to visualize each and every hepatocyte in the zebrafish liver throughout the entire post-embryonic growth period" were referred to the results reported in Figs 1 and 2, in which we did achieve both visualizing and enumerating each and every hepatocyte in the zebrafish liver across key stages of post-embryonic growth period. However, we can see the statement may be misleading, and have revised it for clarifications.

On page 5, paragraph 2:

"Here, we developed WAO-ExM, aiming to visualize each and every hepatocyte in the zebrafish liver across representative stages of post-embryonic growth period."

4. Example of binculeated hepatocyte in Fig S1 seemed somewhat ambiguous. Perhaps a high mag inset would assist to show distinct nuclei.

Response (3.5): As advised, we have now included a high-magnification inset in S1B Fig to highlight binucleated hepatocytes.

On page 41, S1B Fig:

> "(B) Representative confocal optical sections showing hepatocyte membrane (gray) and nuclei (red) at 7 dpf, 21 dpf, 42 dpf, and adult stages. The yellow arrow and zoomed-in inset highlight a binucleated hepatocyte. Scale bar, 10 μm."
