# Supplementary material for: Whole-organ single-cell mapping defines growth dynamics and clonal organization in developing and adult zebrafish livers
Source: PLoS Biol. 2026 Sep 28;24(9):e3004007. doi: 10.1371/journal.pbio.3004007 (PMC13641717; doi:10.1371/journal.pbio.3004007)
Supplement: Peer review history

# Editor report: Decision Letter 0

**DOI:** 10.1371/journal.pbio.3004007.r001

**Authors:** Ines Alvarez-Garcia

25 Jun 2026

Dear Dr Chen,

Thank you for submitting your manuscript entitled "Whole-organ single-cell mapping defines growth dynamics and clonal organization in developing and adult zebrafish livers" for consideration as a Methods and Resources by PLOS Biology.

Your manuscript has now been evaluated by the PLOS Biology editorial staff as well as by an academic editor with relevant expertise and I am writing to let you know that we would like to send your submission out for external peer review. However, after discussions with the Academic Editor, we would like to consider the manuscript as a Research Article. We think the novelty of the method is undermined by previous studies that used similar techniques to visualise large tissue regions. Thus, please select that type of article from the drop down menu.

Before we can send your manuscript to reviewers, we need you to complete your submission by providing the metadata that is required for full assessment. To this end, please login to Editorial Manager where you will find the paper in the 'Submissions Needing Revisions' folder on your homepage. Please click 'Revise Submission' from the Action Links and complete all additional questions in the submission questionnaire.

Once your full submission is complete, your paper will undergo a series of checks in preparation for peer review. After your manuscript has passed the checks it will be sent out for review. To provide the metadata for your submission, please Login to Editorial Manager (https://www.editorialmanager.com/pbiology) within two working days, i.e. by Jun 29 2026 11:59PM.

If your manuscript has been previously peer-reviewed at another journal, PLOS Biology is willing to work with those reviews in order to avoid re-starting the process. Submission of the previous reviews is entirely optional and our ability to use them effectively will depend on the willingness of the previous journal to confirm the content of the reports and share the reviewer identities. Please note that we reserve the right to invite additional reviewers if we consider that additional/independent reviewers are needed, although we aim to avoid this as far as possible. In our experience, working with previous reviews does save time.

If you would like us to consider previous reviewer reports, please edit your cover letter to let us know and include the name of the journal where the work was previously considered and the manuscript ID it was given. In addition, please upload a response to the reviews as a 'Prior Peer Review' file type, which should include the reports in full and a point-by-point reply detailing how you have or plan to address the reviewers' concerns.

During the process of completing your manuscript submission, you will be invited to opt-in to posting your pre-review manuscript as a bioRxiv preprint. Visit http://journals.plos.org/plosbiology/s/preprints for full details. If you consent to posting your current manuscript as a preprint, please upload a single Preprint PDF.

Feel free to email us at plosbiology@plos.org if you have any queries relating to your submission.

Kind regards,

Ines

--

Ines Alvarez-Garcia, PhD

Senior Editor

PLOS Biology

ialvarez-garcia@plos.org

# Peer review: Decision Letter 1

**DOI:** 10.1371/journal.pbio.3004007.r002

**Authors:** Ines Alvarez-Garcia

30 Jul 2026

Dear Dr Chen,

Thank you for your patience while your manuscript entitled "Whole-organ single-cell mapping defines growth dynamics and clonal organization in developing and adult zebrafish livers" went through peer-review at PLOS Biology. Your manuscript has now been evaluated by the PLOS Biology editors, an Academic Editor with relevant expertise, and by two independent reviewers.

The reviews are attached below. As you will see, the reviewers are very positive, raising only minor points. Reviewer 1 offers several suggestions for improving regarding the interpretation of the developmental growth burst, quantification and interpretation of dominant clones, the laminin phenotype and intestinal morphogenesis, statistics and the analysis of the liver cancer model. Reviewer 2 mentions a few points, mostly requiring further discussion or missing information.

In light of the reviews, we are pleased to offer you the opportunity to address the comments from the reviewers in a revision that we anticipate should not take you very long. In addition, the Academic Editor thinks you should soften the claim of a new method because imaging of larger tissue (like the brain) has already been done, and citing those additional papers that performed larger tissue imaging would be helpful.

We will then assess your revised manuscript and your response to the reviewers' comments with our Academic Editor aiming to avoid further rounds of peer-review, although we might need to consult with the reviewers, depending on the nature of the revisions. In addition to these revisions, you may need to complete some formatting changes, which you will receive in a follow up email. A member of our team will be in touch with a set of requests shortly. If you do not receive a separate email within a few days, please assume that checks have been completed, and no additional changes are required.

We expect to receive your revised manuscript within 1 month. Please email us (plosbiology@plos.org) if you have any questions or concerns, or would like to request an extension.

At this stage, your manuscript remains formally under active consideration at our journal; please notify us by email if you do not intend to submit a revision so that we withdraw the manuscript.

**IMPORTANT - SUBMITTING YOUR REVISION**

Your revisions should address the specific points made by each reviewer. Please submit the following files along with your revised manuscript:

1. A 'Response to Reviewers' file - this should detail your responses to the editorial requests, present a point-by-point response to all of the reviewers' comments, and indicate the changes made to the manuscript.

*NOTE: In your point-by-point response to the reviewers, please provide the full context of each review. Do not selectively quote paragraphs or sentences to reply to. The entire set of reviewer comments should be present in full and each specific point should be responded to individually.

You should also cite any additional relevant literature that has been published since the original submission and mention any additional citations in your response.

2. In addition to a clean copy of the manuscript, please also upload a 'track-changes' version of your manuscript that specifies the edits made. This should be uploaded as a "Revised Article with Changes Highlighted " file type.

3. Resubmission Checklist

When you are ready to resubmit your revised manuscript, please refer to this resubmission checklist: https://plos.io/Biology_Checklist

To submit a revised version of your manuscript, please go to https://www.editorialmanager.com/pbiology/ and log in as an Author. Click the link labelled 'Submissions Needing Revision' where you will find your submission record.

Please make sure to read the following important policies and guidelines while preparing your revision and fulfil the editorial requests:

a) *PLOS Data Policy*

Please note that as a condition of publication PLOS' data policy (http://journals.plos.org/plosbiology/s/data-availability) requires that you make available all data used to draw the conclusions arrived at in your manuscript. If you have not already done so, you must include any data used in your manuscript either in appropriate repositories, within the body of the manuscript, or as supporting information (N.B. this includes any numerical values that were used to generate graphs, histograms etc.). Please also indicate in each figure legend where the data can be found. For an example see here: http://www.plosbiology.org/article/info%3Adoi%2F10.1371%2Fjournal.pbio.1001908#s5

b) *Published Peer Review*

Please note while forming your response, if your article is accepted, you may have the opportunity to make the peer review history publicly available. The record will include editor decision letters (with reviews) and your responses to reviewer comments. If eligible, we will contact you to opt in or out. Please see here for more details:

https://blogs.plos.org/plos/2019/05/plos-journals-now-open-for-published-peer-review/

c) *Blot and Gel Data Policy*

Please provide the original, uncropped and minimally adjusted images supporting all blot and gel results reported in an article's figures or Supporting Information files. We will require these files before a manuscript can be accepted so please prepare them now, if you have not already uploaded them. Please carefully read our guidelines for how to prepare and upload this data: https://journals.plos.org/plosbiology/s/figures#loc-blot-and-gel-reporting-requirements

d) *Protocols deposition*

To enhance the reproducibility of your results, we recommend that if applicable you deposit your laboratory protocols in protocols.io, where a protocol can be assigned its own identifier (DOI) such that it can be cited independently in the future. For instructions see: https://journals.plos.org/plosbiology/s/submission-guidelines#loc-materials-and-methods

Thank you again for your submission to our journal. We hope that our editorial process has been constructive thus far, and we welcome your feedback at any time. Please don't hesitate to contact us if you have any questions or comments.

Sincerely,

Ines

--

Ines Alvarez-Garcia, PhD

Senior Editor

PLOS Biology

ialvarez-garcia@plos.org

----------------------------------------------------------------

Reviewers' comments

Rev. 1: Sumeet Pal Singh – note that this reviewer has signed the review.

Roan et al. present the development and application of whole adult-organ expansion microscopy (WAO-ExM) for single-cell-resolved imaging of intact zebrafish organs. The authors combine tissue clearing, expansion microscopy, light-sheet imaging and large-volume image analysis to quantify hepatocyte numbers across post-embryonic liver growth. They further integrate this approach with clonal lineage tracing, genetic perturbation of laminin function and analyses of additional organs.

The principal strength of the study is the exceptionally high-quality imaging. The whole-organ reconstructions are visually impressive and provide a clear demonstration of the technical capabilities of WAO-ExM. In particular, the preservation of cellular resolution across millimetre-scale samples and through considerable tissue depth is convincing. The images of the developing and adult liver, the multicolour clonal analysis and the extension of the method to the heart and pancreas are of excellent quality. The accompanying image-processing workflow should also be valuable to laboratories working with large volumetric datasets.

The biological observations are interesting. The quantitative analysis identifies a pronounced increase in hepatocyte number between 21 and 42 dpf and confirms that post-embryonic liver growth is associated with substantial heterogeneity in clonal contribution. The finding that individual clones can occupy a large fraction of the ventral lobe is particularly striking. The laminin experiments further suggest that changes in tissue morphology can occur without a comparable change in total hepatocyte number.

Overall, this is a technically strong and carefully performed study that should be of broad interest to researchers working in developmental biology, organ growth and quantitative imaging. The manuscript is generally well written, although several conclusions would benefit from more cautious interpretation and some additional methodological clarification.

Major comments

1. Interpretation of the developmental growth burst

The large increase in hepatocyte number between 21 and 42 dpf is an important observation. However, the present data consist of relatively widely separated developmental time points. The conclusion that liver growth occurs through a sharply defined proliferative burst may therefore be somewhat stronger than the temporal resolution permits. The authors could either moderate this wording or, where feasible, include one or two intermediate time points. Or include quantification of cell proliferation using EdU or Ki67 / PCNA.

Even without additional experiments, it would be appropriate to describe this interval as a period of markedly accelerated hepatocyte accumulation rather than implying that the precise timing and duration of the proliferative phase have been fully resolved.

2. Quantification and interpretation of dominant clones

The clonal analysis is visually compelling and is one of the strongest parts of the manuscript. However, the estimate of cell number within each labelled clone is based on DAPI-positive nuclei contained within a GFP-defined volume. As the authors acknowledge, this measurement may include endothelial, immune, biliary and other non-hepatocyte nuclei.

The cell-number estimates should therefore be described more explicitly as the number of nuclei contained within the clonal territory rather than the exact number of hepatocytes in the clone. Alternatively, the authors could restrict the analysis to nuclei positive for a hepatocyte-specific nuclear marker. This distinction is important because the estimated clone sizes are used to support the conclusion that individual hepatocytes generate more than 10,000 descendants.

The manuscript should also clarify how confidently a large, uniformly coloured territory can be assigned to a single original hepatocyte. Although sparse induction substantially reduces the likelihood of adjacent cells independently acquiring similar colours, Brainbow reporters do not necessarily provide a unique barcode for every labelled cell. The possibility of similarly labelled neighbouring clones should be addressed.

3. Laminin phenotype and intestinal morphogenesis

The lamb1a experiment provides an interesting example of organ deformation without a major change in total hepatocyte number. However, the data do not yet establish that laminin acts directly within the liver to determine liver shape. Lamb1a has broad developmental functions, and the temperature-sensitive perturbation is systemic.

Importantly, the post-embryonic remodelling of zebrafish liver morphology occurs in close physical association with intestinal bending and folding. The abnormal liver shapes observed after lamb1a inactivation could therefore arise secondarily from altered intestinal morphogenesis, changes in intestinal folding or altered mechanical constraints imposed by surrounding tissues. The authors acknowledge this possibility in the Discussion, but it should be given greater prominence when interpreting the results.

Accordingly, statements such as "laminins regulate liver growth by defining tissue shape" or that liver shape and hepatocyte proliferation are independently regulated should be moderated. The present data show that systemic lamb1a inactivation alters liver morphology without measurably changing total hepatocyte number. Determining whether this is a liver-autonomous ECM phenotype or an indirect consequence of abnormal intestinal folding will require tissue-specific perturbation or simultaneous analysis of intestinal morphology.

It would be useful to show the liver together with the adjacent intestine in control and mutant animals, where possible. Even a descriptive comparison of intestinal bending and liver positioning would help readers assess this alternative explanation.

4. Statistical power in the lamb1a analysis

The whole-organ imaging of the lamb1a mutants is impressive, but the quantitative analysis includes six controls and four mutants. In addition, only approximately one-third of mutant animals developed severe liver abnormalities. It is not entirely clear whether the four animals used for hepatocyte counting were selected based on morphological phenotype.

The authors should clarify the selection criteria and report the individual morphological category for each quantified liver. A comparison between morphologically affected mutants, unaffected mutants and sibling controls would be more informative than grouping all mutants together. With the current sample size, the absence of a statistically significant difference should not be interpreted as proof that hepatocyte number is unchanged. Reporting an effect-size estimate and confidence interval would make the conclusion more precise.

5. Analysis of the liver cancer model

The application of WAO-ExM to the cancer model demonstrates the potential utility of the method. However, this section is relatively preliminary. The reduction in total hepatocyte number is accompanied by reduced body length, and the p-value is close to the conventional significance threshold. The suggestion that the phenotype reflects competition between proliferative cancer cells and normal hepatocytes is speculative and is not directly tested.

The authors should substantially moderate this interpretation. Reduced hepatocyte number could reflect developmental delay, altered differentiation, cell death, changes in reporter expression or broader effects of the transgenic and p53-mutant background. The section would be stronger if it were presented primarily as a proof-of-principle application of WAO-ExM to diseased tissue.

The manuscript should also clarify whether the fabp1a:H2A-mCherry reporter remains uniformly expressed in transformed hepatocytes. If reporter expression is reduced or heterogeneous in the cancer model (due to dedifferentiation), hepatocyte counting could underestimate the total cell population.

Minor comments

1. The manuscript alternates between fabp10a and lfabp when describing the reporter. The nomenclature should be standardised.

2. The statement that adult zebrafish liver contains an average of 1,265,206 "cells" should specify "hepatocytes," since the liver also contains several non-parenchymal cell types.

3. Sex should be reported where known, particularly for adult liver measurements. Sexual dimorphism and reproductive status may influence liver size and hepatocyte number.

4. Figure 1 contains visible tile-to-tile intensity variation. This does not undermine the cellular resolution, but the authors could comment on whether intensity normalisation or illumination correction was applied before segmentation.

5. The apparent reuse or inversion of labels for the supplementary movies should be checked carefully. The Results and figure legends should be cross-checked against the uploaded supplementary files.

6. In the Methods, the authors state that identical detection parameters were used across all datasets. Given the different expansion factors and acquisition systems, it would be helpful to specify whether "identical" refers to physical dimensions after correction for expansion or to raw pixel-based parameters.

7. For the pancreas analysis, the authors quantify ins-reporter-positive nuclei as beta cells. The manuscript should briefly state whether smaller secondary islets were included or excluded from the reported count.

8. "Heterogenicity" should be changed to "heterogeneity."

9. "Compliment histological assays" should be changed to "complement histological assays."

10. "Dox and Tam ware administered" should be corrected to "were administered."

11. The Methods state that actb was used as the qPCR reference gene, whereas Table S1 lists primers for rpl13a. This discrepancy should be corrected.

12. The manuscript refers to "Lamb1a inactivation" and, elsewhere, more broadly to "laminin inactivation." Since the experiment specifically perturbs *lamb1a*, the wording should remain gene-specific unless loss of the broader laminin complex has been demonstrated.

Rev. 2:

This beautiful and important study makes a significant contribution to our understanding of post-embryonic development and emergence of adult form, in examining cellular composition and arrangements in the liver of zebrafish from early larva to adult stages, using expansion microscopy and light-sheet imaging. The study also examines morphology of the heart, another important model for regenerative biology, and cancerous tissue, and assess clonal contributions to liver composition using a new "liverbow" transgenic system . It is fair to say the work raises the bar for what can be accomplished using these preparative and imaging modalities and will almost surely stimulate new work to better understand these kinds of phenomena across a wider range of tissues and organ systems, as well as organisms. I have only a few comments:

1. Review copy lacked Movie S1. Probably fine but check for errors in call outs etc.

2. Authors use chronological age as their metric for describing developmental progress but the relationship between age and stage varies considerably across labs. For this study, 21 and 42 dpf are especially important waypoints but for some labs a 42 dpf fish will really be the same stages as a 21 dpf fish. So while dpf is certainly easy to understand and can be a useful point of reference relative to how the work flow actually proceeded, the authors need to address the potential for age/developmental stage disparity and provide a rough estimate of the true fish stages they examined at their chosen time points (and genotypes). This all likely merits no more than a sentence someplace early in the results or in a figure legend, and stages corresponding to ages can be provided in just the one place, using mm Standard Length, which is highly correlated with true developmental state across a variety of traits, retaining dpf if preferred elsewhere once its true meaning has been clarified. Note this applies also to the tumor-bearing fish that are smaller at 21 dpf; in this case an indication of size alone might be insufficient as the fish could be either developmentally at an earlier state, or they could be severely growth-deficient while nevertheless attaining the same developmental state as controls (in which case other described indicators of development stage could be reported). These kinds of benchmarks will add to rigor and reproducibility for the current study, but also work derived from it, especially important now for exploiting the true potential of the authors' methods and their own enabling of quantitative analyses.

3. Results from analyzing clonal changes in composition are very intriguing. The descriptions should, however, enumerate how many individuals were examined, repeatedly, across each of the stages and how many of those were subjected to terminal expansion microscopy at the end. If most or all were single-stage analyses then the trend still seems clear but the introductory goal "to visualize each and every hepatocyte in the zebrafish liver throughout the entire post-embryonic growth period" could be misleading with respect to actual outcomes.

4. Example of binculeated hepatocyte in Fig S1 seemed somewhat ambiguous. Perhaps a high mag inset would assist to show distinct nuclei.

# Author comment: Author response to Decision Letter 2

**DOI:** 10.1371/journal.pbio.3004007.r003

18 Aug 2026

Attachment Submitted filename: Roan et al_response letter.pdf

# Editor report: Decision Letter 2

**DOI:** 10.1371/journal.pbio.3004007.r004

**Authors:** Ines Alvarez-Garcia

4 Sep 2026

Dear Dr Chen,

Thank you for the submission of your revised Research Article entitled "Whole-organ single-cell mapping defines growth dynamics and clonal organization in developing and adult zebrafish livers" for publication in PLOS Biology. On behalf of my colleagues and the Academic Editor, Cody Smith, I am delighted to let you know that we can in principle accept your manuscript for publication, provided you address any remaining formatting and reporting issues. These will be detailed in an email you should receive within 2-3 business days from our colleagues in the journal operations team; no action is required from you until then. Please note that we will not be able to formally accept your manuscript and schedule it for publication until you have completed any requested changes.

Please take a minute to log into Editorial Manager at http://www.editorialmanager.com/pbiology/, click the "Update My Information" link at the top of the page, and update your user information to ensure an efficient production process.

PRESS

We frequently collaborate with press offices. If your institution or institutions have a press office, please notify them about your upcoming paper at this point, to enable them to help maximise its impact. If the press office is planning to promote your findings, we would be grateful if they could coordinate with biologypress@plos.org. If you have previously opted in to the early version process, we ask that you notify us immediately of any press plans so that we may opt out on your behalf.

We also ask that you take this opportunity to read our Embargo Policy regarding the discussion, promotion and media coverage of work that is yet to be published by PLOS. As your manuscript is not yet published, it is bound by the conditions of our Embargo Policy. Please be aware that this policy is in place both to ensure that any press coverage of your article is fully substantiated and to provide a direct link between such coverage and the published work. For full details of our Embargo Policy, please visit http://www.plos.org/about/media-inquiries/embargo-policy/.

Many congratulations and thanks again for choosing PLOS Biology for publication and supporting Open Access publishing. We look forward to publishing your study.

Sincerely,

Ines

--

Ines Alvarez-Garcia, PhD

Senior Editor

PLOS Biology

ialvarez-garcia@plos.org
